# Playing with Structural Parameters: Synthesis and Characterization of Two New Maltol-Based Ligands with Binding and Antineoplastic Properties

**DOI:** 10.3390/molecules25040943

**Published:** 2020-02-20

**Authors:** Eleonora Macedi, Daniele Paderni, Mauro Formica, Luca Conti, Mirco Fanelli, Luca Giorgi, Stefano Amatori, Gianluca Ambrosi, Barbara Valtancoli, Vieri Fusi

**Affiliations:** 1 Department of Pure and Applied Sciences, University of Urbino “Carlo Bo”, via della Stazione 4, 61029 Urbino, Italy; daniele.paderni@uniurb.it (D.P.); mauro.formica@uniurb.it (M.F.); luca.giorgi@uniurb.it (L.G.); gianluca.ambrosi@uniurb.it (G.A.); 2 Department of Chemistry “Ugo Schiff”, University of Florence, via della Lastruccia 3-13, 50019 Sesto Fiorentino, Italy; luca.conti@unifi.it (L.C.); barbara.valtancoli@unifi.it (B.V.); 3 Department of Biomolecular Sciences, Molecular Pathology Laboratory “PaoLa”, University of Urbino “Carlo Bo”, via Arco d’Augusto 2, 61032 Fano, Italy; mirco.fanelli@uniurb.it (M.F.); stefano.amatori@uniurb.it (S.A.)

**Keywords:** antitumor agents, ligand design, maltol, metallo-receptors, N ligands

## Abstract

Two maltol-based ligands, *N*,*N*′-bis((3-hydroxy-4-pyron-2-yl)methyl)-1,4-piperazine (**L1**) and *N*,*N*′,*N*′-tris((3-hydroxy-4-pyron-2-yl)methyl)-*N*-methylethylendiamine (**L2**), were synthesized and characterized. **L1** and **L2**, containing, respectively, two and three maltol units spaced by a diamine fragment, were designed to evaluate how biological and binding features are affected by structural modifications of the parent compound malten. The acid-base behavior and the binding properties towards transition, alkaline-earth (AE) and rare-earth (RE) cations in aqueous solution, studied by potentiometric, UV-Vis and NMR analysis, are reported along with biological studies on DNA and leukemia cells. Both ligands form stable complexes with Cu(II), Zn(II) and Co(II) that were studied as metallo-receptors for AE and RE at neutral pH. **L1** complexes are more affected than **L2** ones by hard cations, the **L1**-Cu(II) system being deeply affected by RE. The structural modifications altered the mechanism of action: **L1** partially maintains the ability to induce structural alterations of DNA, while **L2** provokes single strand (nicks) and to a lesser extent double strand breaks of DNA.

## 1. Introduction

Cancer has long been known and still is one of the most common diseases, despite the great technological and social development we have experienced in the last few decades. The rise in incidence is alarming: a 61.7% worldwide increase of cases by 2040 is predicted [1].

Overall, the number of cancer survivors has grown, but even if this trend shows that progress is being made against the disease, much work remains [2].

Often, the survival of patients is limited by the lack of specific therapies; the cytotoxicity of the drugs, and the resulting occurrence of severe side effects; and, not least, the resistance to currently used chemotherapics. In that light, finding new antineoplastic drugs with reduced toxicity and relevant clinical activity is currently a major therapeutic challenge.

In the search for new molecules providing high and selective therapeutic anticancer efficacy, while being well tolerated by the human body, one important strategy is to look at natural sources.

**Figure 1:**
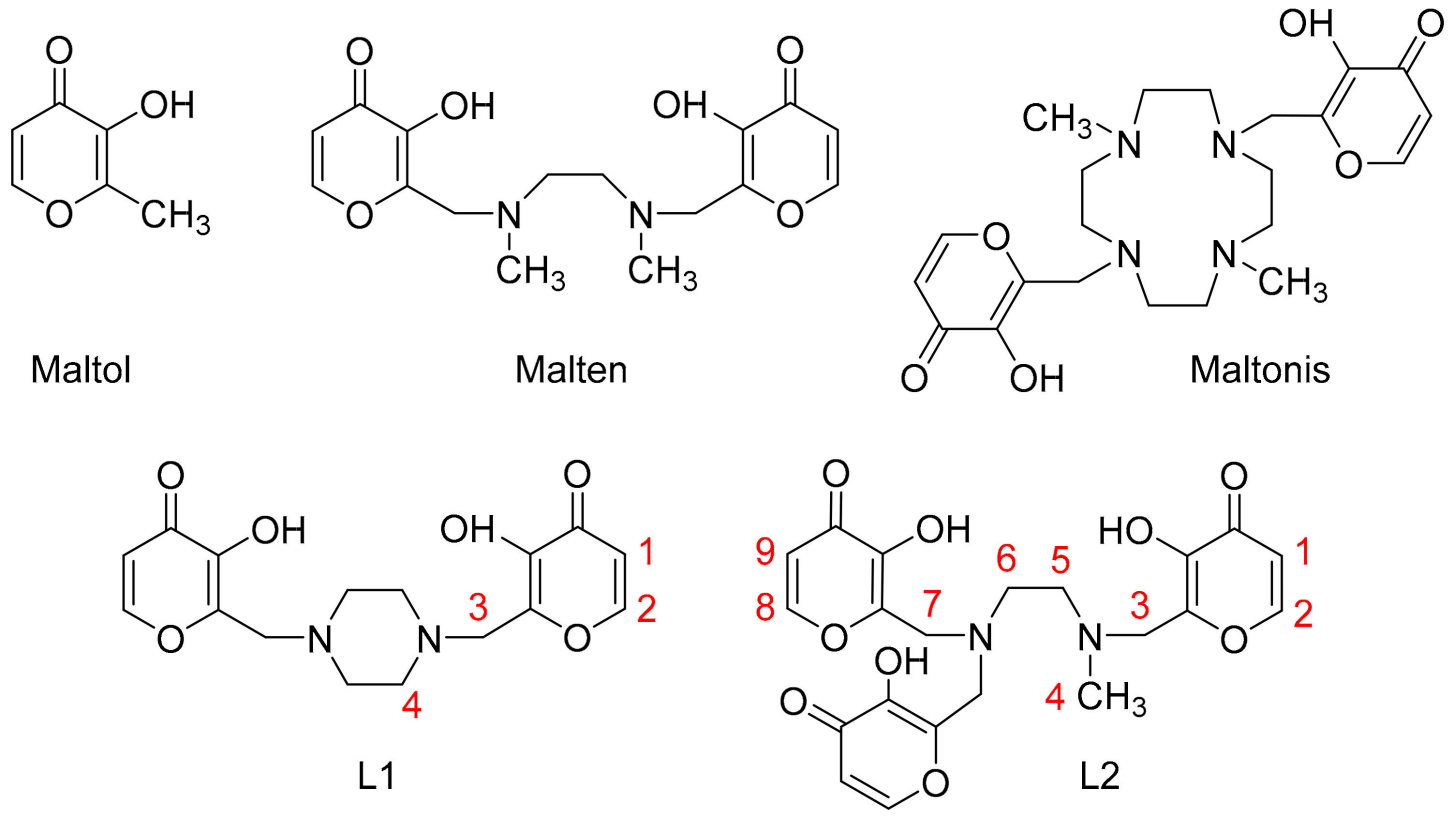
Compounds maltol, malten, maltonis, **L1** and **L2** with atom labelling used in NMR assignments.

Maltol (3-hydroxy-2-methyl-4-pyrone, Figure 1) is a naturally occurring substance, having been long and widely used as a safe and reliable flavoring agent, food preservative and natural antioxidant.

Besides its common application as a flavor potentiator [3], maltol has been used in catalysis, and for cosmetic and pharmaceutical formulations [4,5,6,7].

It is known that a ROS scavenger can be used in the treatment of diseases such as anemia, tumor, nerve cell oxidative stress and diabetes-induced irreversible kidney damage [8,9,10,11]. As a growth inhibitor, maltol can be combined effectively with free radicals of body [12]; moreover, the ROS scavenging and antioxidant properties of maltol provide the molecule with antineoplastic [13,14,15,16], neuroprotective [17,18] and anti-apoptotic activities [19], and make it able to attenuate acute alcohol-induced liver injury and prevent oxidative damage in mice [20,21,22].

In addition, maltol possesses coordination properties towards metal ions: maltol-derived organometallic complexes have potential antitumor activity [9,23] and ligands containing maltol have been developed and exploited as new metal-based antitumor drugs [24,25,26,27]. In addition, oxovanadium(IV) complexes of maltol-based ligands were developed as insulin enhancing agents [28,29].

Due to its acid-base properties and biocompatibility [28,29], maltol is a good choice in the development of biologically active compounds.

Linear and macrocyclic polyamines, generally with symmetrical topology, are also known antitumor agents [30,31,32]. Furthermore, they show well-known coordination properties towards metal ions and/or anions [33,34,35]. Due to their protonable sites, polyamines allow one to obtain water-soluble compounds; that is a crucial point for both biological activity and guest coordination in aqueous solutions.

Thus, aimed both at discovering new drugs for treating cancer and at synthesizing ligands provided with marked binding properties towards metal ions, we recently developed a new class of maltol-based ligands by symmetrically coupling polyamines and maltol units, which exhibited antineoplastic activity in vitro [36,37,38,39] and in vivo [40]. The compounds show two (3-hydroxy-4-pyron-2-yl)methyl units separated by an aliphatic amine spacer, which could be either linear, as in the case of *N*,*N*′-bis((3-hydroxy-4-pyron-2-yl)methyl)-*N*,*N*′-dimethylethylendiamine (malten, Figure 1), or cyclic, as in the case of 4,10-bis[(3-hydroxy-4-pyron-2-yl)methyl]-1,7-dimethyl-1,4,7,10-tetraazacyclododecane (maltonis, Figure 1).

Malten, by cell-free assay, was monitored to increase the complexity of the genomic DNA structure through the possible induction of DNA intermolecular crosslinking [38]. The treatment with malten causes a dose-dependent reduction in cell survival in all the neoplastic models studied, associated with the activation of cell cycle arrest, and finally, the programmed cell death (apoptosis). Moreover, malten exposure modulates the expression of genes having key roles in cell cycle progression and apoptosis [36,37,38,39].

Maltonis is more effective than malten, and noteworthily, is effective on sarcoma (in vivo) and multidrug- and cisplatin-resistant cells (in vitro) while being ineffective on normal human mesenchymal stem cells. Its mechanism of action seems to involve the inhibition of cell proliferation and the involvement of a DNA damage response till induction of cell death [40].

Finally, both ligands possess the ability to induce covalent binding between DNA and proteins, suggesting a molecular mechanism of action that may involve the interference with the chromatin structure [39,40]. Interestingly, the simultaneous presence of two amino-spaced maltol units seems to be the key of the biological activity of these molecules.

The two polyaza ligands show acid-base properties, their protonation degree and thus the biologically active species depending on the pH value. In particular, the main species present in solution at pH 7.4 are the zwitterionic form **L** and the H**L**^+^ cation for malten and maltonis, respectively [36,37,38,39].

Besides the biological activity, both ligands show marked coordination properties toward metal cations. Indeed, they are able to bind first row transition metal ions such as Cu(II) and Co(II), forming preorganized 1:1 complex species which are in turn able to bind hard metal cations of the alkaline, alkaline-earth and rare-earth series in a 2:1 ratio [41,42,43]. In particular, the copper(II) complex of malten, thanks to the preorganization of the receptor, exhibits the interesting ability both to distinguish between Ca^2+^ and Mg^2+^ and to bind cations of the rare earth series, targets that are generally difficult to achieve in aqueous solution [41,42].

Following these results, in this contribution we expanded our research to other maltol-derived ligands, playing with factors such as the stiffness of the polyamine scaffold and the number of maltol units. The objective of the present work is indeed the design of new ligands for the purpose of modifying the parent compound malten to evaluate how the peculiar binding properties in an aqueous solution and the biological activity are affected by specific structural modifications. The flexibility of malten was claimed to be a crucial property for the interaction with DNA [39]; therefore, both a more stiff structure and an increased steric hindrance, which may restrict the conformational freedom of the molecule, affect the binding properties of the molecule, on one hand, and possibly prevent the DNA structural alterations observed for malten, on the other hand.

Likewise, malten, the new ligand *N*,*N*′-bis((3-hydroxy-4-pyron-2-yl)methyl)-1,4-piperazine (**L1**, Figure 1), possesses two maltol units similarly spaced but a more rigid structure which could account for a higher stability in physiological environment, and as a consequence, a slower degradation time. This may eventually lead to a better antineoplastic activity. The ligand *N*,*N*′,*N*′-tris((3-hydroxy-4-pyron-2-yl)methyl)-*N*-methylethylendiamine (**L2**, Figure 1), instead, possesses an additional maltol unit with respect to the previous ligands malten and maltonis, and is therefore a more encumbered and asymmetric molecule.

The two ligands were synthesized and characterized, and both acid-base and stability studies were performed by potentiometric, UV-VIS and NMR measurements. The coordination properties of both ligands in an aqueous solution towards some first row transition, alkaline-earth (AE) and rare-earth (RE) metal ions were also studied by potentiometric and UV-VIS measurements. Finally, herein we report the preliminary in vitro biological studies of both ligands.

## 2. Results and Discussion

### 2.1. Synthesis

**Scheme 1:**
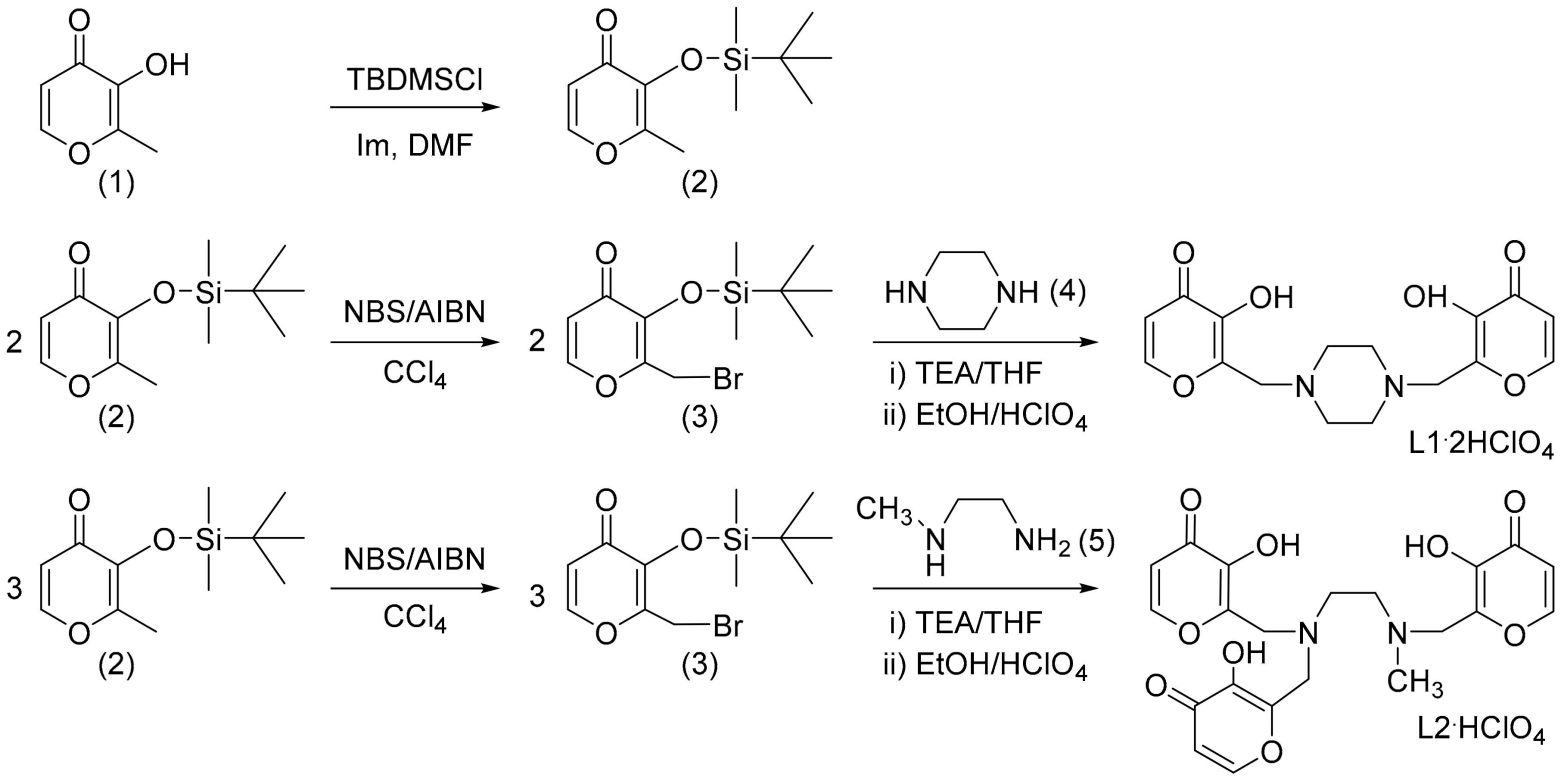
Synthetic pathway to obtain **L1** and **L2**.

The two compounds *N*,*N*′-bis((3-hydroxy-4-pyron-2-yl)methyl)-1,4-piperazine (**L1**) and *N,N*′,*N*′-tris((3-hydroxy-4-pyron-2-yl)methyl)-*N*-methylethylendiamine (**L2**) were synthesized following procedures analogous to those adopted for malten and maltonis [39], which are reported in Scheme 1. Compound **3** was synthesized starting from the commercially available 2-methyl-3-hydroxy-4-pyrone (maltol, **1** in Scheme 1) [44,45]. The commercial compounds **4** and **5** were used without any further purification.

In the synthetic scheme of both ligands (Scheme 1), maltol (**1**) was reacted sequentially with tert-butyldimethylsilyl chloride (TBDMSCl) and *N*-bromosuccinimide to first protect the OH group (**2**), and then activate the methyl group as bromide (**3**). Without any further purification, two or three equivalents of **3** freshly prepared were then coupled with **4** and **5**, respectively, to obtain the crude products **L1** and **L2**. The crude products were then added dropwise of a 10% perchloric acid ethanolic solution to precipitate the desired compounds as hydroperchlorate salts. The acid treatment is aimed both at removing the protective tert-butyldimethylsilyl groups and cleansing the compounds, thereby avoiding time-consuming and expensive chromatographic purifications. Finally, the two products were easily obtained as white solids by recrystallization from a saturated sodium perchlorate aqueous solution (**L1**) or by washing with warm acetonitrile (**L2**).

### 2.2. Acid-base Behavior

The protonation constants of **L1** and **L2** were potentiometrically determined in 0.15 mol dm^−3^ NaCl, for **L1**, and NMe_4_Cl, for **L2**, aqueous solutions at 298.1 K and are summarized in Table 1.

Under the investigated experimental conditions, **L1** behaves as a diprotic acid, whereas **L2** behaves as a triprotic acid, because of the additional maltol unit; both are diprotic bases.

The ligands exist in alkaline aqueous solution (pH ≥ 10) in the fully deprotonated form (H_−2_**L**^2−^ and H_−3_**L**^3−^ for **L** = **L1** and **L2**, respectively) which is able to add up to four or five protons (for **L1** and **L2**, respectively) to convert into the fully protonated H_2_**L**^2+^ species at acidic pH (pH < 2).

The protonation constants of **L1** and **L2** for the same protonation degree are very similar to each other; **L2** possesses an additional maltol with respect to **L1**; indeed, it shows a supplementary constant featuring the highest value. In comparison to the protonation constants of malten (log *K*_1−4_: 9.03, 7.86, 6.24, 3.17), **L1** and **L2** show very similar but lower values for the same protonation degree, revealing similar basicity.

Besides such analogies with malten, **L1** and **L2** show similar acid-base properties. The protonation values in both cases exhibit a linear decrease in the basicity up to the addition of the penultimate proton, and then a sharp decrease in the last step is observable.

Such a trend accounts for an easy accessibility of the protonation sites up to the H**L**^+^ species, and on the contrary, a quite unfavorable last protonation step, due to the electrostatic repulsion. It can be supposed, thus, that the acidic protons in the H**L**^+^ species are most likely located as far as possible from each other; namely, on the maltolate moieties and on an amine function. The value for the first proton addition (log *K*_1_ = 8.67 and 9.46 for **L1** and **L2**, respectively) is similar to the first protonation constant of the free 1,4-dimethylpiperazine (log *K* = 8.38) [46] and *N,N*′-dimethylethylendiamine (log *K* = 10.16) [47], suggesting that it occurs on an amine function.

The following protonation steps probably involve the maltolate moieties (log *K*_2−3_ = 7.77, 5.88 for **L1** and *K*_2−4_ = 8.33, 7.37, 5.85 for **L2**) being similar to the basicity of the free deprotonated maltol (log *K* = 8.44) [48], as previously observed for malten.

The last protonation involving the remaining amine group drops the constant value due to electrostatic repulsion between the two close ammonium groups (log *K*_4_ = 2.28 and log *K*_5_ = 1.86 for **L1** and **L2**, respectively).

**Figure 2:**
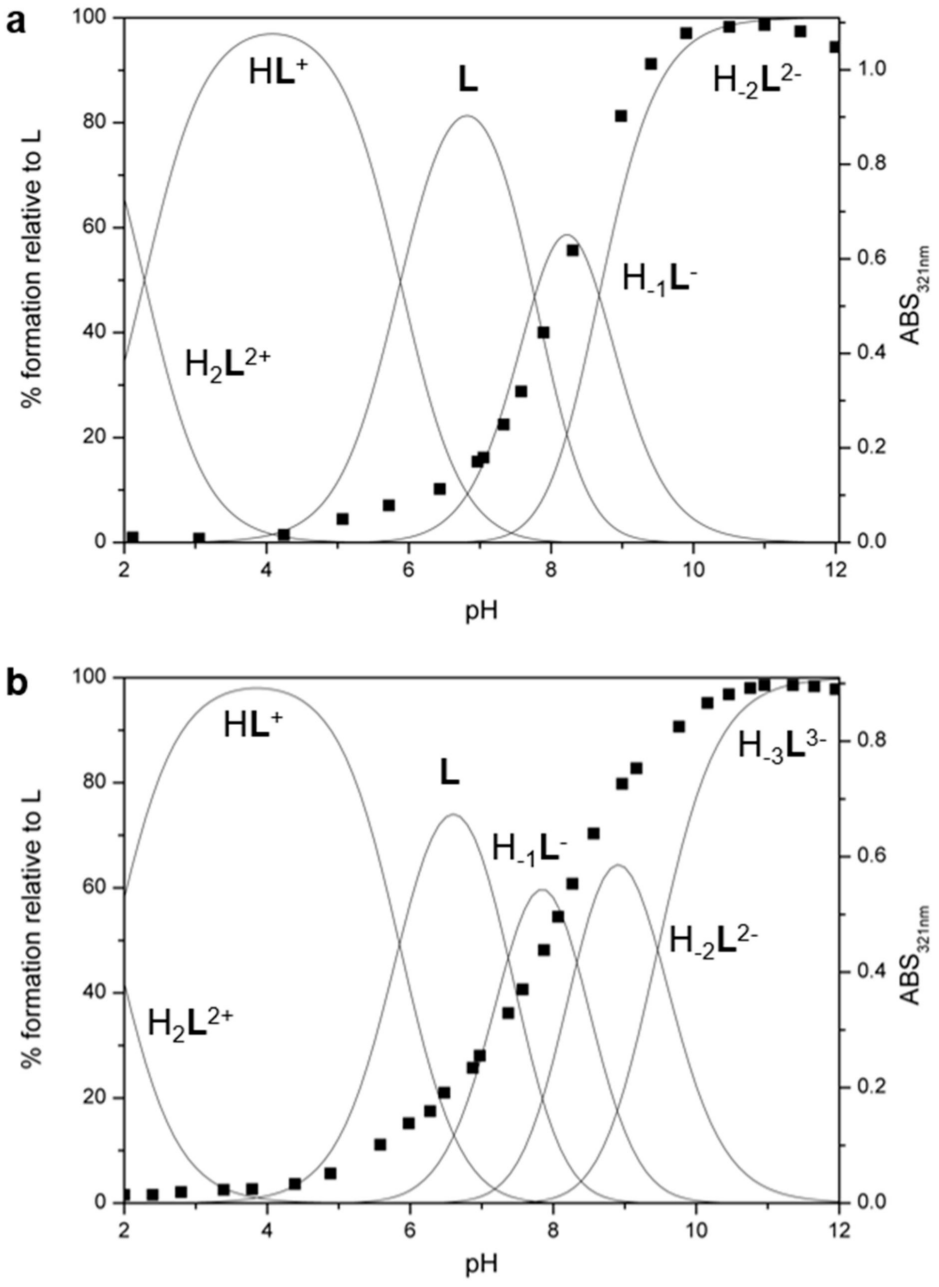
Distribution diagram of the species (−) and trend of the absorbance (■) for (**a**) **L1** and (**b**) **L2** (λ = 321 nm) in aqueous solution as a function of pH. UV-Vis measurements: [**L1**] = [**L2**] = 4.4 × 10^−5^ mol dm^−3^; potentiometric measurements: [**L1**] = [**L2**] = 1 × 10^−3^ mol dm^−3^, I = 0.15 mol dm^−3^ NaCl and NMe_4_Cl for **L1** and **L2**, respectively, T = 298.1 K.

Figure 2 shows the distribution diagrams of the protonated species of **L1** and **L2** together with the trend of the absorbance at 321 nm in aqueous solutions as a function of pH (*vide infra*).

#### UV-VIS and ^1^H–NMR Studies

UV-VIS absorption electronic measurements and ^1^H–NMR analysis as a function of pH were performed on both ligands to investigate the distributions of protons in the species present in solution at different pH values.

UV-VIS analysis shows, for both ligands, a similar behavior. At pH 2, where the fully protonated H_2_**L**^2+^ species is present in solution, they exhibit a band at λ*_max_*= 275 nm (ε = 30500 and 25400 cm^−1^ mol^−1^ dm^3^ for **L1** and **L2**, respectively). Upon increasing the pH, the band at 275 nm declines until disappearance, whereas a new band at λ*_max_* = 321 nm (ε = 24900 and 20400 cm^−1^ mol^−1^ dm^3^ for **L1** and **L2**, respectively) appears and reaches its maximum at pH = 10 and 11 for **L1** and **L2**, respectively. The two bands can be ascribed to the neutral (275 nm) or deprotonated (321 nm) form of the maltol group.

The absorbance of the deprotonated maltolate species is visible in the spectra from pH > 4, where the neutral **L** species start to be present in solution, and keeps increasing up to pH = 10 for **L1** and pH = 11 for **L2**, where the H_−1_**L1**^−^ and H_−2_**L2**^2−^ species are fully formed (Figure 2).

This behavior, in agreement with the potentiometric studies, suggests that the first and last protonation steps occur on amine functions, whereas the remaining steps occur on the maltolate groups.

**Figure 3:**
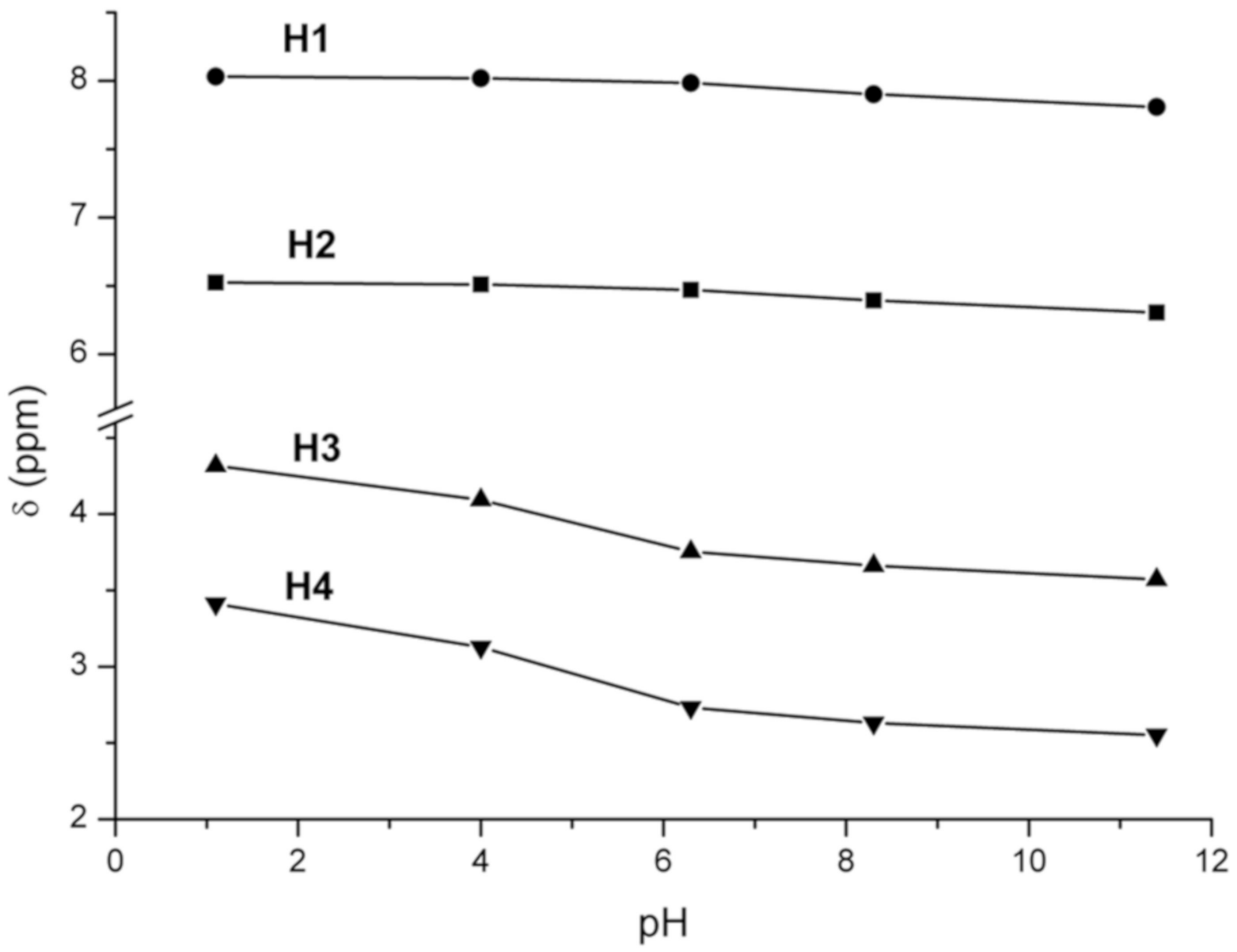
^1^H–NMR chemical shift of **L1** in an aqueous solution as a function of pH.

In Figure 3 the trend for the chemical shifts of the ^1^H–NMR resonances of **L1** as a function of pH is reported, while that for **L2** is reported in Figure S1 (see Figure 1 for atom labelling used in NMR assignments).

Analyzing **L1** (Figure 3), starting from alkaline pH and considering the pH range 11.5–8.5, where the H_−2_**L1**^2−^ and H_−1_**L1**^−^ species are present in solution, the aliphatic signals (H3, H4) show a little downfield shift coupled with a slight downfield shift of the aromatic protons (H1, H2); this trend suggests that the first protonation step mainly involves an amine function, giving rise to the formation of a hydrogen bond involving the closest maltolate group to stabilize the acidic proton.

In the pH range 8.5–6.5, where the **L1** species forms, the resonances of the aromatic protons undergo the largest shift, meaning that the maltolate not involved in H-bonding is being protonated.

In the pH ranges 6.5–4 and 4–1, where the H**L1**^+^ and H_2_**L1**^2+^ species, respectively, are prevalent in solution, mainly the aliphatic signals are involved, undergoing a marked downfield shift. In the former pH range, results suggest that also the second maltolate is being protonated, disrupting the H-bond and increasing the electron density on the nitrogen atom, thereby perturbing the resonances of the neighboring protons. Finally, in the latter pH range the last protonation step involves the remaining amine site.

^1^H–NMR resonances of **L2** exhibit similar behavior, leading to a comparable discussion (see Supporting Information and Figure S1).

**Figure 4:**
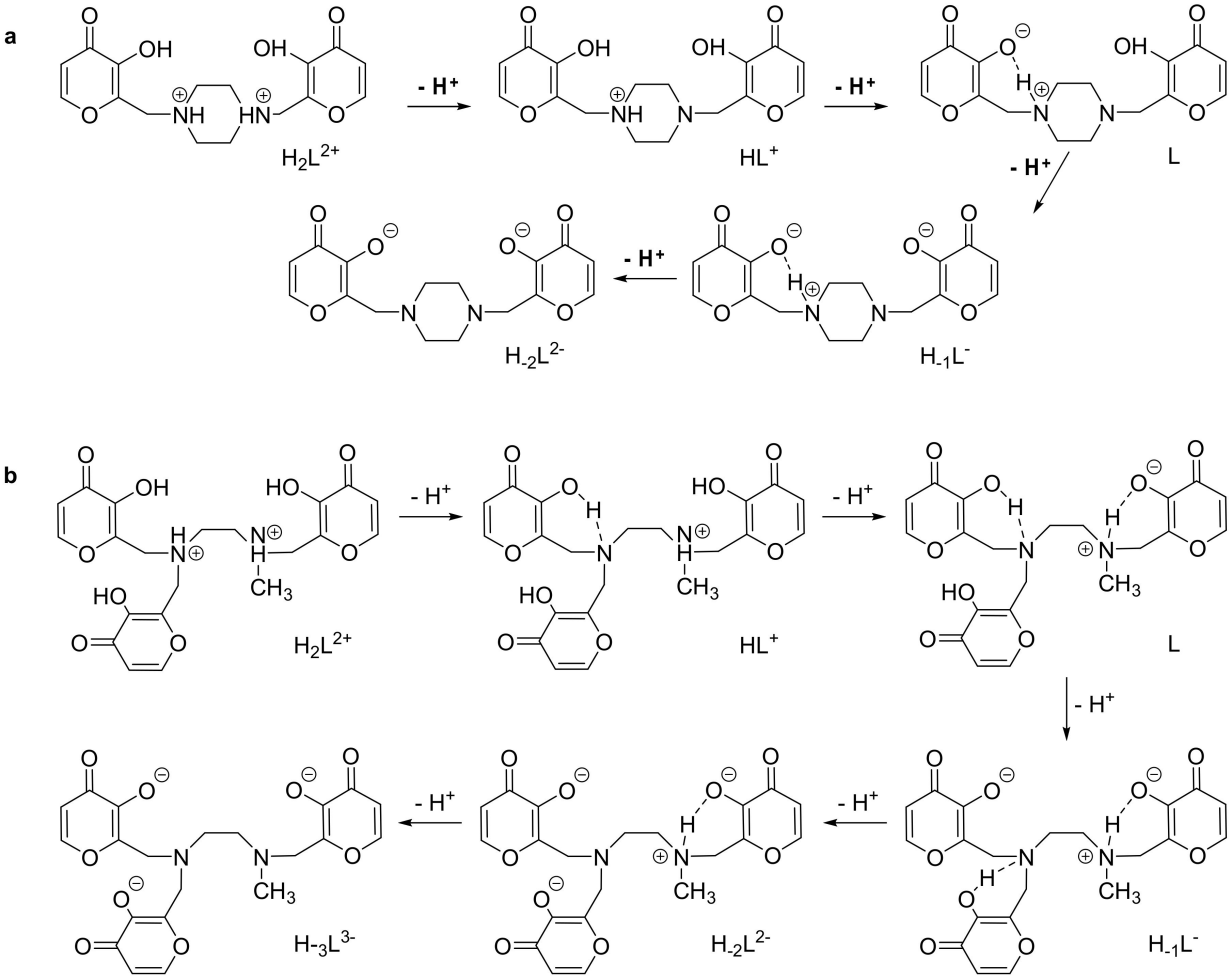
Proposed location of acidic hydrogen atoms in the protonated species of (**a**) **L1** and (**b**) **L2**.

Merging the analysis of potentiometric, UV-VIS and ^1^H–NMR data, it is possible to suggest protonation schemes for **L1** and **L2** highlighting the probable location of the acid protons in the several protonated species (Figure 4).

In both cases, two species are present at the physiological pH 7.4—the zwitterionic form **L** and the anionic form H_−1_**L**^−^, which can be considered as the species present in the biological medium at such a pH.

### 2.3. Stability

The stabilities of **L1** and **L2** in an aqueous solution at the physiological pH 7.4 were studied by means of UV-VIS and ^1^H–NMR spectroscopies, to eventually understand the mode and timing of the degradation processes of the ligands.

To this purpose, buffered solutions of the two ligands were prepared at pH 7.4 (4-(2-hydroxyethyl)-1-piperazine-ethanesulphonic acid—HEPES, pKa = 7.55), to make sure that pH stays unchanged with temperature and time.

UV-VIS measurements performed over a period of five days (120 h) suggest a far higher decomposition for **L2** than for **L1** at pH 7.4 (more details in the Supporting Information, Figure S2).

Nevertheless, UV-VIS measurements gave only qualitative data regarding the stability of the two ligands. To find out more conclusive data about stability and determine the time required for 50% degradation of **L1** and **L2** at the physiological pH 7.4, ^1^H–NMR experiments were performed (Figures S3 and S4). In this case, the phosphoric buffer was used instead of HEPES, to not have the superimposition of HEPES signals with those of the examined ligand.

The integration of peak areas allowed us to calculate the degradation times for **L1** and **L2**. The latter is in line with that previously reported for malten, the time required for 50% degradation being 10 h for both ligands, whereas **L1** shows a much longer timing of 48 h.

The overall data suggest for **L2,** higher and faster degradation than for **L1** at pH 7.4.

### 2.4. Coordination of Metal Ions

The binding properties of **L1** and **L2** towards Cu(II), Zn(II) and Co(II) transition metal ions were investigated by potentiometric measurements in 0.15 mol dm^−3^ NaCl and NMe_4_Cl for **L1** and **L2**, respectively, in aqueous solution at 298.1 K.

**Figure 5:**
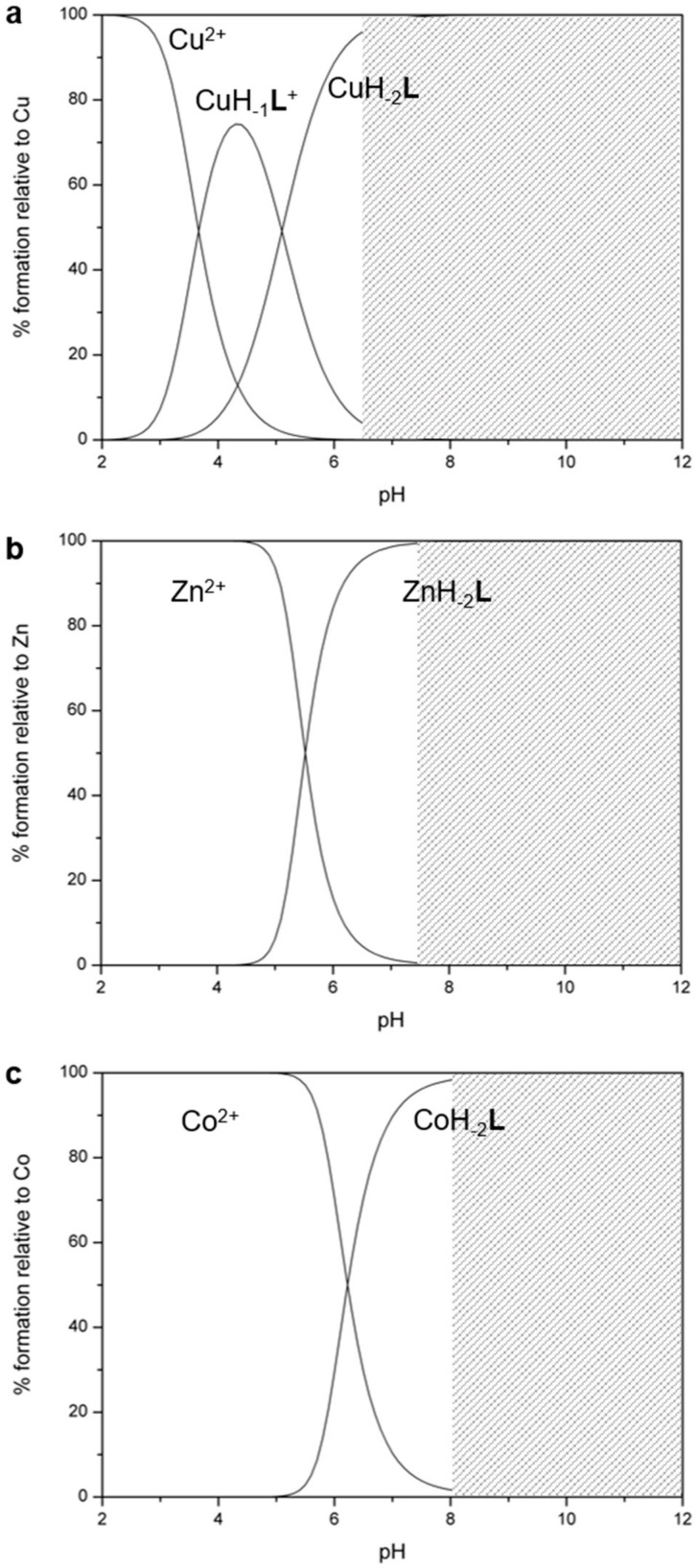
Distribution diagram of the species for the (**a**) **L**-Cu(II), (**b**) **L**-Zn(II) and (**c**) **L**-Co(II) (**L** = **L1**) system in 1:1 molar ratio as a function of pH in an aqueous solution (I = 0.15 mol dm^−3^ NaCl, T = 298.1 K, [**L**] = [Cu(II)]/ [Zn(II)]/ [Co(II)] = 1 × 10^−3^ mol dm^−3^). The scarce solubility of the neutral species of **L1** with all metal cations precluded the investigation beyond pH 6.3, 7.3 and 8.0 for Cu(II), Zn(II) and Co(II), respectively, under these experimental conditions. Accordingly, dark areas have been inserted in the graphs.

**Figure 6:**
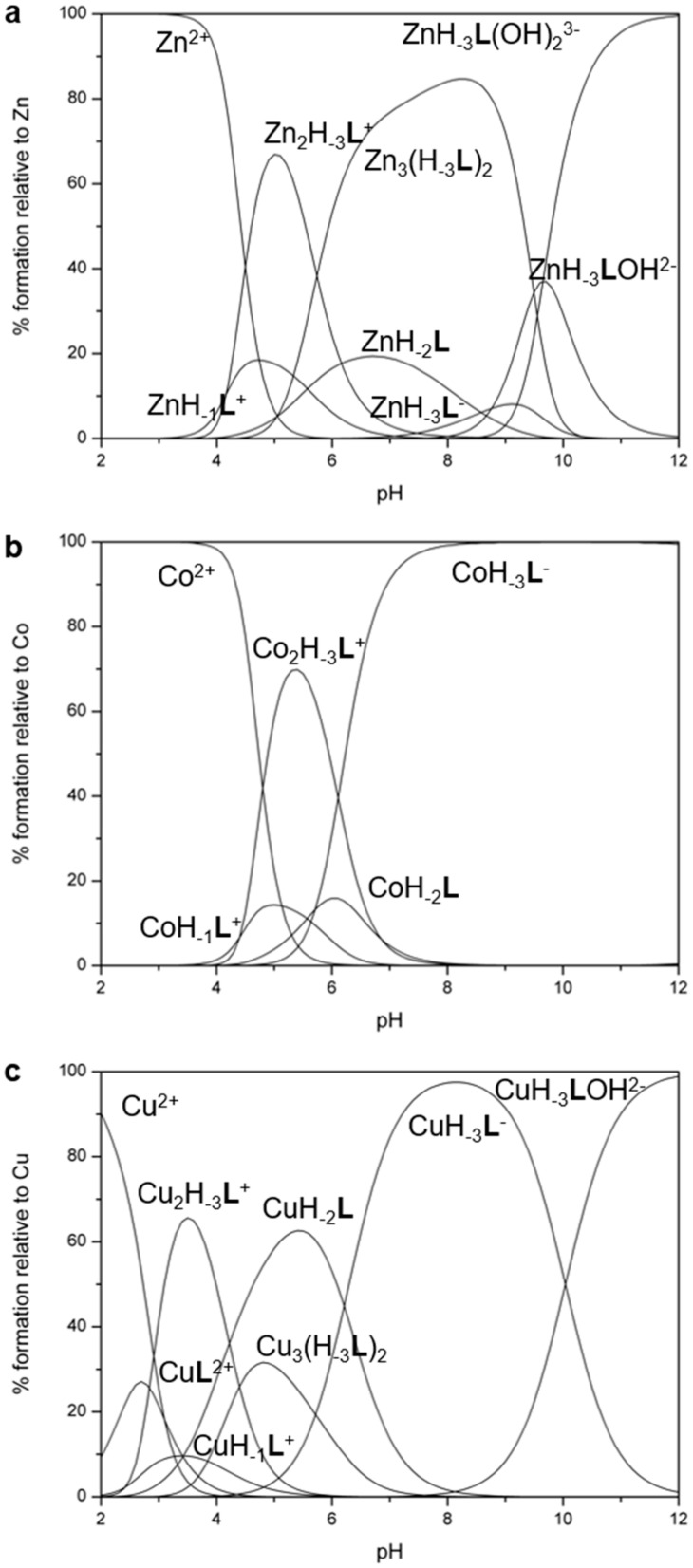
Distribution diagram of the species for the (**a**) **L**-Zn(II), (**b**) **L**-Co(II) and (**c**) **L**-Cu(II) (**L** = **L2**) system in 1:1 molar ratio as a function of pH in an aqueous solution (I = 0.15 mol dm^−3^ NMe_4_Cl, T = 298.1 K, [**L**] = [Zn(II)]/ [Co(II)]/ [Cu(II)] = 1 × 10^−3^ mol dm^−3^).

The stability constants for the complexation reactions of **L1** and **L2** with such cations are reported in Table 2 and Table 3. The same measurements were performed for the system malten-M(II) (M(II) = Zn(II) and Co(II)) and are reported for comparison (Table 2). The malten-Cu(II) system was previously reported [41]. Figure 5 and Figure 6 report the distribution diagrams of the species for the **L**-M(II) systems (**L** = **L1**, **L2**; M(II) = Cu(II), Zn(II), Co(II)) as a function of pH (see Figure S5 for diagrams relative to malten-M(II) (M(II) = Zn(II), Co(II); for the malten-Cu(II) system, see reference [41]).

#### 2.4.1. Mononuclear Complexes

**L1**, **L2** and malten are able to form mononuclear complexes with all the metal ions investigated, whose stabilities are consistent with the Irving-Williams series, the constant values being higher for Cu(II) than for Co(II) and Zn(II) in all cases (Table 2 and Table 3).

Contrarily to **L2**, **L1** and malten can only form mononuclear complexes; indeed, species with different stoichiometry were not detected in these experimental conditions (Figure 5 and Figure S5; for the malten-Cu(II) system, see reference [41]). In the case of **L1**, the species was prevalent in solution at pH > 6 with all metal ions as [M(H_−2_**L**)]. The corresponding malten species was present in solution at pH > 6, in the case of Zn(II) and Co(II), while [Cu(H_-2_**L**)] prevails in a wider range of pH (4–11).

**L1** forms mononuclear [M(H_−2_**L**)] species of lower stability than malten with all the metal ions investigated (log *K* = 13.22, 9.22, 7.46 vs. log *K* = 17.53 [41], 10.29, 10.35 for Cu(II), Zn(II) and Co(II), respectively). This can be attributed to the stiffening of the **L1** molecular framework ascribable to the 1,4-piperazine compared to the 1,2-dimethylendiamine fragment in malten. The hexacyclic scaffold not only is more rigid but disfavors the involvement of both maltol functions in the metal ion coordination, which indeed requires the conformational change of the ring from chair to boat with subsequent loss of energy; this justifies the lower stability constants for **L1** compared to malten. In any case, an analogous coordination environment around the M(II) ion of both ligands could be hypothesized. Based on the crystal structures of malten [41,42], two oxygen atoms of the deprotonated maltol functions of **L1** along with two nitrogen atoms of the polyamine are probably involved in the coordination of the M(II) ion in a N_2_O_2_ environment.

Only the [Cu(H_−2_**L1**)] species is able to add one proton, whereas the mononuclear [M(H_−2_**L**)] species of malten are able to add up to two protons in the case of Cu(II), and one proton for the Zn(II) and Co(II) species. In all cases, the addition constant values agree with the involvement of sites already engaged in the coordination of the metal ion. In particular, the [Cu(H_−2_**L**)] species of malten ends to be the less prone to be protonated.

Finally, contrarily to the mononuclear complexes of **L1**, those of malten are all able to form the monohydroxylated [M(H_−2_**L**)OH]^−^ species with very similar constant values (Table 2).

Unfortunately, the scarce solubility of the neutral complex species of **L1** with all the tested metal ions precluded the investigation beyond pH 6.3, 7.3 and 8.0 for Cu(II), Zn(II) and Co(II), respectively, under these experimental conditions.

The mononuclear species of **L2** prevail in solution for a metal to ligand ratio of 1:1, except in the case of Zn(II) (Figure 6).

The comparison between the formation constants of the corresponding mononuclear species of **L2** and malten (log *K* = 18.25, 10.74, 12.88 vs. log *K* = 17.53 [41], 10.29, 10.35 for Cu(II), Zn(II) and Co(II), respectively) suggests for Cu(II) and Zn(II), an analogous coordination environment around the metal cations in the two ligands. In particular, based on the corresponding values for malten and on the crystal structure of the malten-Cu(II) complex [42], the involvement of two maltolate functions of **L2** can be hypothesized in the coordination of Cu(II) and Zn(II), while all three maltolate functions seem to be involved in the coordination of Co(II), due to the higher constant value with respect to the malten cobalt complex. Thus, it can be reasonably hypothesized that the cation is coordinated by two nitrogen atoms of the polyamine and by two or three oxygen atoms of the deprotonated maltol functions in a N_2_O_2_ (for Cu(II) and Zn(II)) or N_2_O_3_ (for Co(II)) environment.

As in the case of malten, **L2** does not offer an effective stabilization of the **L**-Zn(II) and **L**-Co(II) systems, as maltonis instead does (log *K* = 18.78 for Co(II)) [43], most likely due to the lower number of donor atoms in **L2** and the different coordination geometries offered by the ligands.

The mononuclear complexes of **L2** can add up to two protons in the case of Zn(II) and Co(II) complexes, or three protons in the case of the Cu(II) complex; the addition constant values confirm the previous statement and suggest that in the case of Co(II), both protonation steps involve sites already engaged in the coordination of the metal ion. In other words, all three maltolate functions seem to be involved in the coordination of Co(II).

As for Cu(II) and Zn(II) complexes, the values for the addition of the first proton suggest the involvement of a free site, the values being similar to that for free maltol [48], confirming the hypothesis that only two of the three maltolate functions are involved in the coordination. The following lower protonation values support the involvement of sites already engaged in the coordination of the metal ion.

Finally, the mononuclear complexes of **L2** with Cu(II) and Zn(II) are able to form hydroxylated species. The [Zn(H_-3_**L**)]^−^ species is able to form both a mono- and a dihydroxylated species with similar formation constants, whose values are higher than that for the monohydroxylated [Cu(H_−3_**L**)OH]^2−^ species (Table 3). The **L**-Co(II) system does not form any hydroxylated species, once again supporting that all maltolate functions are involved in the coordination of Co(II), satisfying the coordination sphere of Co(II) which is unable to accept external ligands.

#### 2.4.2. Polynuclear Complexes

Among this series of ligands, **L2** is the only one able to form binuclear complexes with all tested metal ions. Such species are prevalent in solution for a metal to ligand ratio of 2:1 (Figures S6a, S7a, S8).

The values for the addition of the second metal cation are quite high, suggesting the formation of stable binuclear complexes with all M(II) cations. The values are, however, different depending on the metal ion; thus, a different coordination environment could be hypothesized for the three cations. In all cases, the [M_2_(H_−3_**L**)]^+^ species is prevalent in solution in a wide range of pH.

Contrarily to the mononuclear species, the binuclear complexes with Cu(II) and Zn(II) do not form hydroxylated species, whereas the [Co_2_(H_−3_**L**)]^+^ cation can add up to two hydroxide ions. The corresponding addition constants are similar and quite low (log *K* = 3.57, 2.47), suggesting that the hydroxide anions are not coordinated in a bridge disposition between the two metal centers.

**L2** is also able to form complexes with a 3:2 stoichiometry involving two units of the fully deprotonated H_−3_**L**^3−^ species and three Cu(II) or Zn(II) ions. The neutral species with [M_3_(H_−3_**L**)_2_] (M = Cu(II), Zn(II)) stoichiometry are predominant in solution (Figures S6b, S7b).

The formation constants are quite high in both cases, with the copper complex being more stable than the zinc complex. Finally, the [Cu_3_(H_−3_**L**)_2_] species forms three hydroxylated species, with addition constants very similar to each other (Table 3).

#### 2.4.3. Interaction with Alkaline-Earth (AE) and Rare Earth (RE) Ions

As previously reported, the parent compound malten is able to form a preorganized 1:1 complex with Cu(II), where the maltolate functions are forced to converge generating an electron-rich pocket that can accommodate a hard cation [41,42]. Following such binding properties, the interaction of **L**-M(II) (**L** = **L1** and **L2**, M(II) = Cu(II), Zn(II) and Co(II)) systems with cations of the alkaline-earth (AE) and rare earth (RE) series was investigated by UV-Vis absorption measurements in aqueous HEPES buffer solution at pH 7.4 (pH 8 in the case of **L1**-Co(II)). In a typical experiment, 0.5 eq. of AE or RE at a time was added to a buffer solution of **L**-M(II) system in a 1:1 molar ratio.

Under these experimental conditions, the [M(H_-2_**L1**)] (M(II) = Cu(II), Zn(II), Co(II)) species is the only one existing in solution; thus, the addition of AE and RE can be interpreted as an addition to such a species. This is also applicable to [M(H_−3_**L2**)]^−^ when M(II) = Cu(II) and Co(II), while in the case of the **L2**-Zn(II) system both [Zn(H_−3_**L2**)]^-^ and [Zn_3_(H_−3_**L2**)_2_] species are present in an approximately 1:4 molar ratio. Nevertheless, **L2**-Zn(II) was tested along with all other systems, revealing that all **L**-Zn(II) complexes, like the **L**-Co(II) ones (**L** = **L1** and **L2**), are not significantly perturbed by the addition of AE cations.

On the contrary, interaction occurs between **L**-Cu(II) systems (**L** = **L1** and **L2**) and Ca(II), Sr(II) and Ba(II), similarly to what was observed for the malten-Cu(II) system [42]. Conversely, Mg(II) does not perturb any of the systems. The interaction with AE ions is more modest for **L2** than for **L1**. In particular, the addition of Ca(II), Sr(II) and Ba(II) to the [Cu(H_−2_**L1**)] complex causes both blueshifts of 6, 5 and 3 nm, respectively, and a decrease of the absorbance of the band at 313 nm, relative to the [Cu(H_−2_**L1**)] complex (Figure S9a). The addition of Ca(II) and Sr(II) to [Cu(H_−3_**L2**)]^−^ causes instead a small 2 nm blueshift and an increase of the absorbance of the band at 303 nm, relative to the [Cu(H_−3_**L2**)]^-^ complex (Figure S9b).

In any case, the selectivity towards Ca(II) vs. Mg(II) of these systems can be underlined, as previously reported for the malten-Cu(II) metallo-receptor [42].

All the **L**-M(II) (**L** = **L1** and **L2**, M(II) = Cu(II), Zn(II), Co(II)) systems are instead affected by the presence of RE ions in solution, **L1** systems being much more perturbed than **L2** systems.

**Figure 7:**
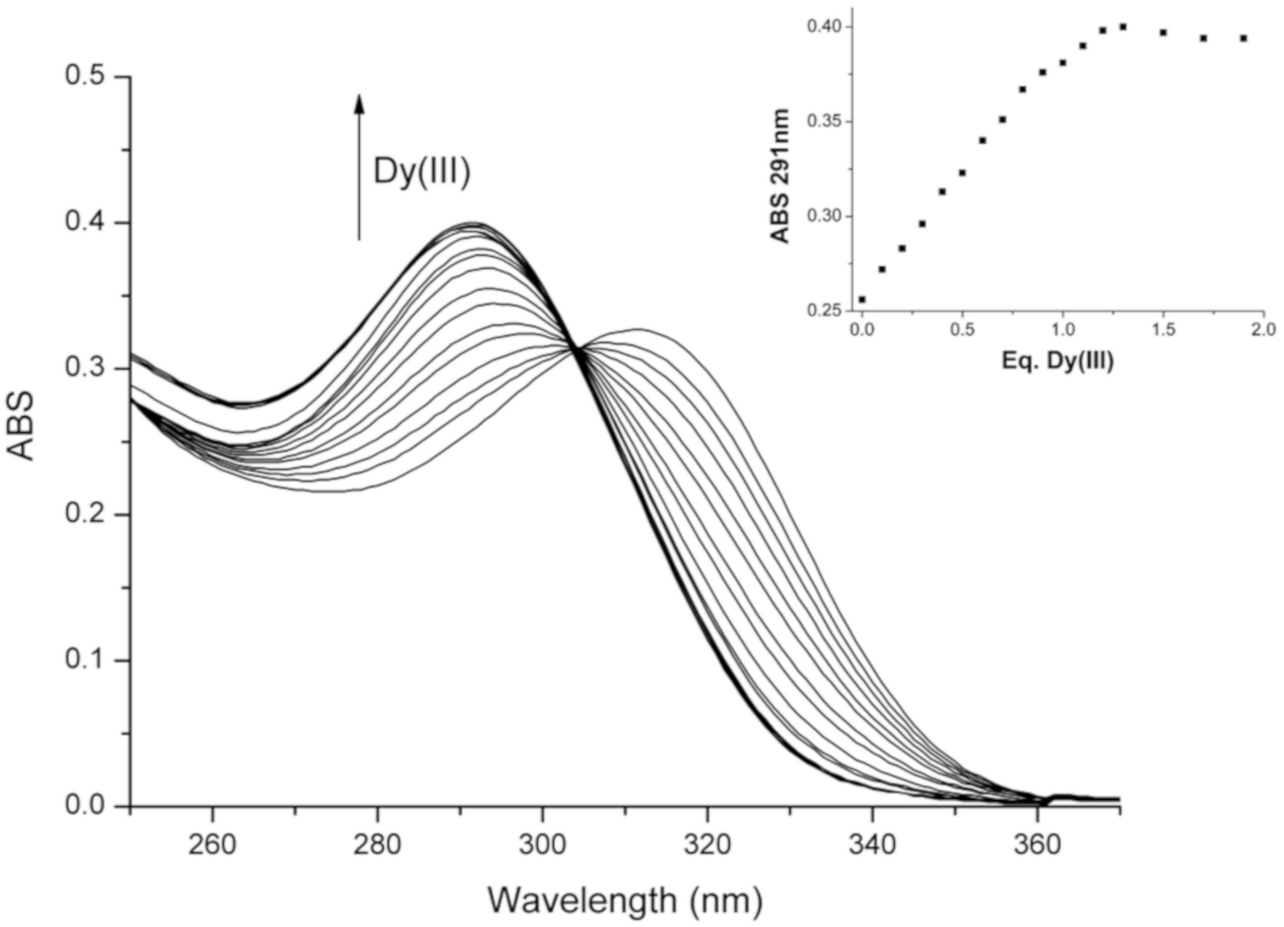
Absorption spectra in the UV-VIS range of the **L1**-Cu(II) system in aqueous solution at pH 7.4 obtained by adding amounts of Dy(III) up to 2 eq. with respect to **L1**-Cu(II) system. [**L1**] = [Cu^2+^] = 2.5 × 10^−5^ mol dm^−3^. Inset: variation of the absorbance at λ = 291 nm as a function of Dy(III) added.

The addition of Eu(III), Dy(III) and Gd(III) to **L1**-M(II) when M(II) = Zn(II) and Co(II), induces a small blueshift or only a slight increase of the absorbance of the band at 313 nm. On the contrary, the addition of RE ions to **L1**-Cu(II) deeply changes the spectrum of the complex, suggesting the strong involvement of the maltolate groups in the RE ion stabilization. The interaction of [Cu(H_-2_**L1**)] with RE was thus investigated more in depth by adding 0.1 eq. of cation at a time. The titration of the copper complex with Dy(III), for example, seems to cause the formation of complexes with different stoichiometries (**L1**-Cu(II):Dy(III) 2:1 and 1:1). The band at 313 nm shifts indeed to 296 nm, and slightly decreases until to the addition of 0.5 eq. of Dy(III); then, the band moves to 291 nm, and the absorbance increases up to the addition of 1 eq. of Dy(III) (Figure 7). The addition of Gd(III) ion to the copper complex of malten produced similar results.

Unfortunately, it was not possible to study the **L1**-Cu(II):RE systems in the visible range to examine the d-d electronic transition band of the copper complex, due to the scarce solubility of the copper complex in the needed concentration.

Moreover, the high stability of the species formed did not allow the safe spectrophotometric determination of the relative stability constants, as occurred similarly for the malten-Cu(II) system. The addition of Eu(III), Dy(III) and Gd(III) to **L2**-M(II) (M(II) = Cu(II), Zn(II) and Co(II)) causes a modest blueshift of the band of the **L2**-M(II) complex and either a small decrease of the absorbance, as was the case of Cu(II) (Figure S10) and Co(II), or an increase of the absorbance, as in the case of Zn(II). Therefore, only a slight interaction between **L2**-M(II) and RE ions can be hypothesized.

### 2.5. Biological Data

**Figure 8:**
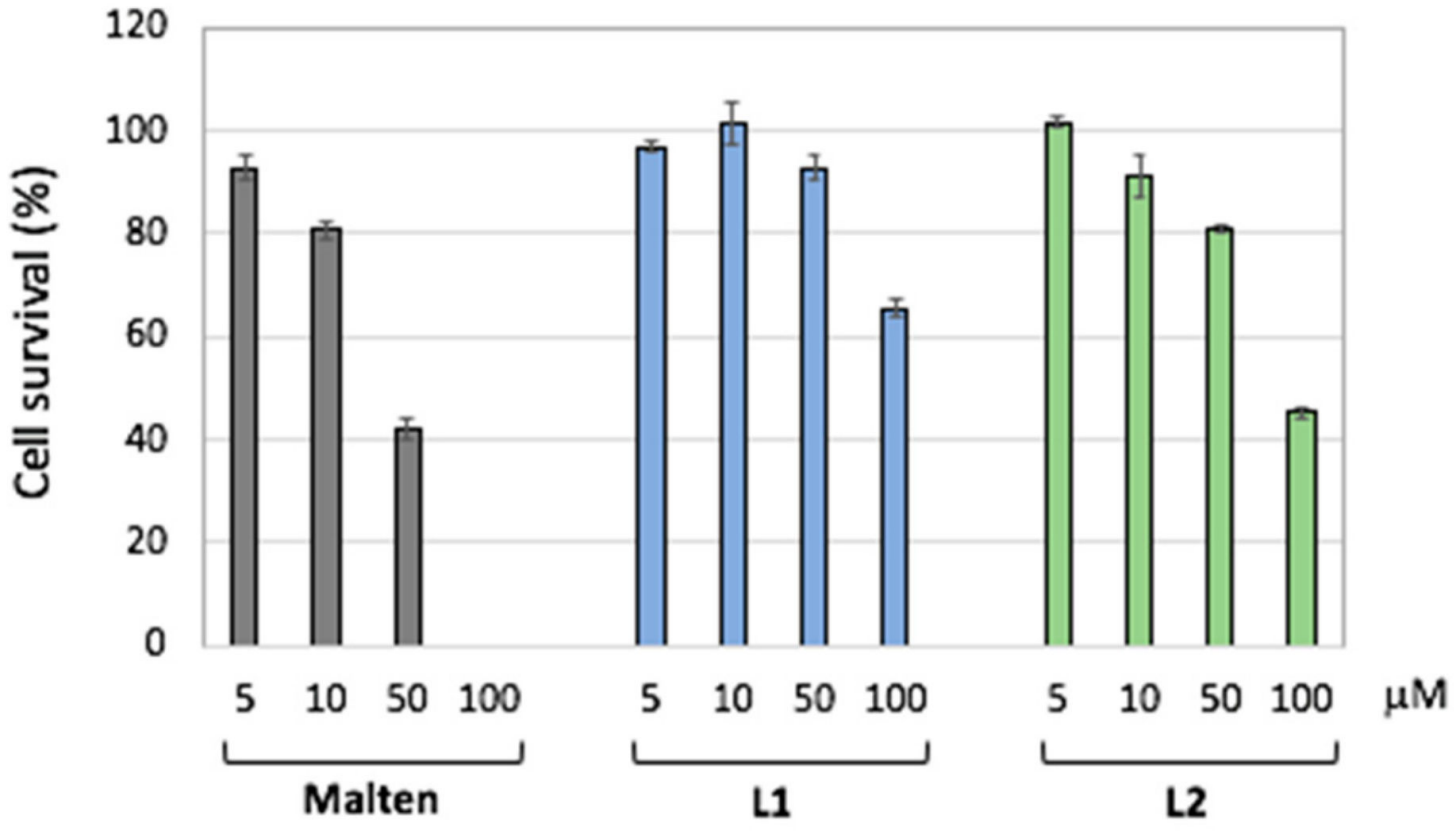
Dose-dependent biological activity of **L1** and **L2**. U937 promonocytic leukemia cell viability was measured by Trypan blue dye exclusion assay (Trypan blue negative cells) after 48 h of treatments. Values are reported as means ± SDs of three independent experiments.

The biological activities of **L1** and **L2** were then investigated by using the promonocytic leukemia (U937) cell line, the same cellular model previously used to test the biological properties of malten [38]. Treatments with ligands **L1** and **L2**—with malten as the reference ligand—at different doses (from 5 µM to 100 µM), were performed in order to evaluate potential biological activity (Figure 8).

Malten was able to reduce the cell survival, as expected, inducing a complete decreased cell survival at the highest dose tested (100 µM), while both **L1** and **L2** showed a lower biological effect at all doses (when compared to malten).

Despite the lower activity monitored, **L2** demonstrated a higher propensity than **L1** to decrease the cell survival of the leukemic cells (Figure 8). The higher effect shown by **L2** with respect to **L1** is also supported by the induction of cell death (26% ± 3.7% Trypan blue positive cells) at 100 µM, while for **L1** no cytotoxicity was monitored. Malten, instead, induced a cytotoxic effect starting at the dose of 50 µM (23% ± 4.6%) reaching the maximum toxicity at 100 µM (82.7% ± 3.3%).

Trying to explain the different biological properties of **L1** and **L2** compared to malten, we molecularly investigated the ability to modify the DNA structure by comparing **L1** and **L2** with the known activity of malten to induce complex structural alterations of a plasmid DNA [38].

**Figure 9:**
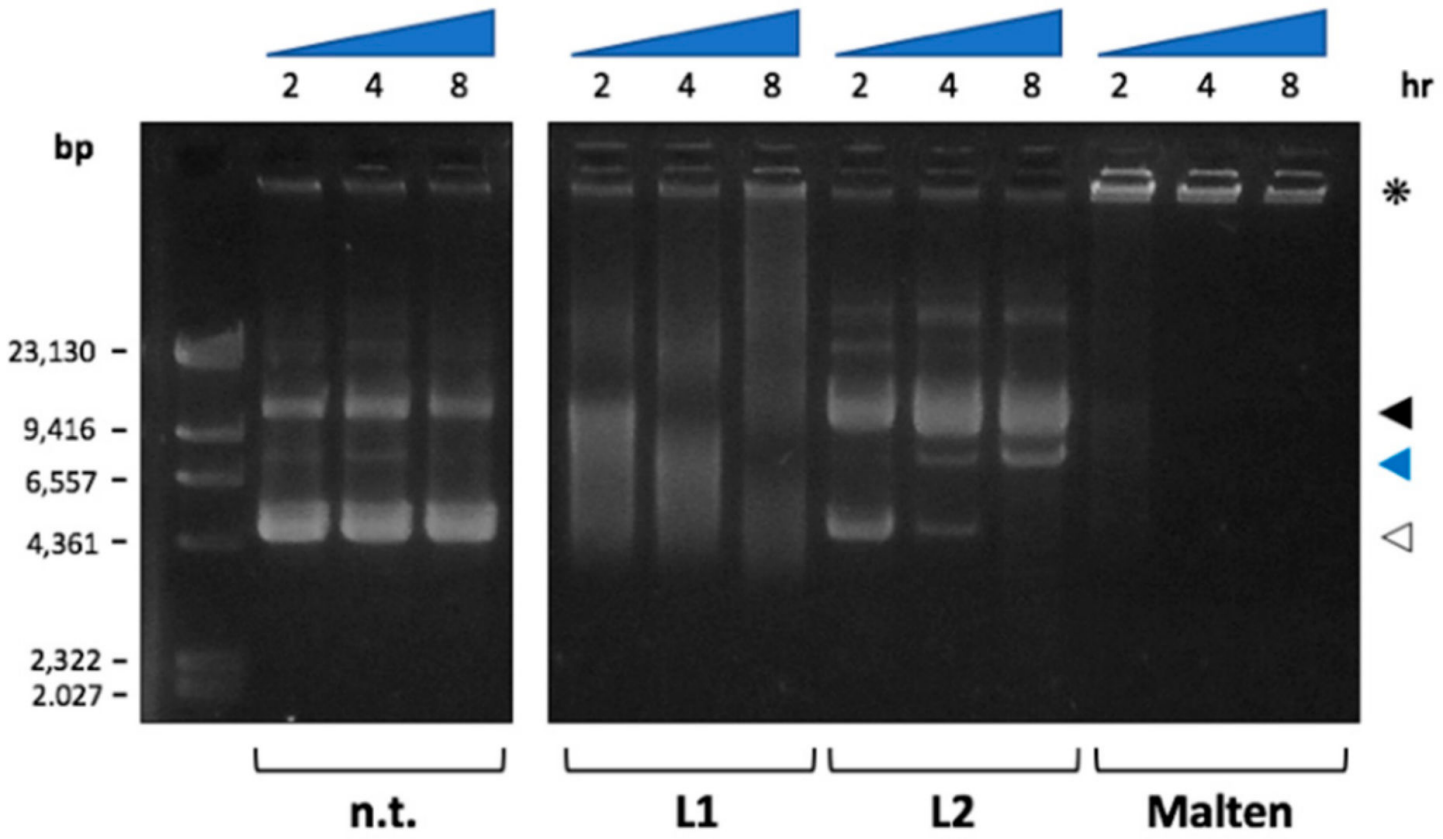
DNA electrophoretic mobility assay. Effects of **L1** and **L2** (malten as control) on the migration of pLL3.7 plasmid DNA. Complex structural alterations (❋), supercoiled DNA (white arrow), open circular DNA (black arrow) and linearized DNA (blue arrow). N.t.—not treated.

Thus, a plasmid DNA (pLL3.7) was first incubated with malten, **L1** and **L2** at different times (2, 4 and 8 h) and then subjected to electrophoretic separation. As reported in Figure 9, **L1** maintains, at least in part, a behavior similar to malten. **L1** is indeed able to induce complex structural alterations of DNA, while being, however, less efficient than the parent compound, as is observable by the amount of non-migrating DNA accumulated in the wells (Figure 9, asterisk). In addition, **L1** is able to induce a smear of DNA that is ascribable to a concomitant DNA degradation.

Interestingly, **L2** has a completely different activity with respect to **L1**; in fact, as shown in Figure 9, **L2** is able to induce a nick of plasmid DNA (single strand break), as demonstrated by the decreased amount of supercoiled form (white arrow) and the concomitant accumulation of the open circular form of the plasmid (black arrow). Furthermore, at late time points (4 and 8 h), the appearance of a linearized form of the plasmid (blue arrow) suggests that **L2** can also induce DNA double strand breaks, without any activity of induction of complex DNA structures similar to those induced by malten, and partially, by **L1**.

## 3. Experimental

### 3.1. UV-Vis and NMR Experiments

UV-Vis absorption spectra were recorded at 298 K on a Varian Cary-100 spectrophotometer (Varian Medical Systems, Crawley, UK) equipped with a temperature control unit.

UV-Vis measurements to evaluate the stability of both **L1** and **L2** were performed in buffer solutions at pH 4, 7.4 and 10, by using, respectively, acetate (pKa = 4.76), HEPES (4-(2-hydroxyethyl)-1-piperazine-ethanesulphonic acid, pKa = 7.55) and CAPS (*N*-cyclohexyl-3-aminopropanesulphonic acid, pKa = 10.4) buffers. Three solutions for each ligand with different pH values were directly prepared in cuvettes ([buffer] = 0.05 mol dm^−3^, [**L**] = 4.4 × 10^−5^ mol dm^−3^), the cuvette then being sealed and stored in the dark at a controlled temperature. The absorbances of the solutions were measured at different interval times over a period of 120 h.

UV-Vis titrations of **L1** and **L2** with M(II) (M(II) = Cu(II), Zn(II), Co(II)) and of **L**-M(II) systems with alkaline earth (AE) and rare earth (RE) ions were performed in aqueous buffer solution (HEPES pH 7.4 in all cases except for the **L1**-Co(II) system, which was studied in HEPES at pH 8) by adding to **L** solutions with increasing amounts of M(II) ions up to 3.0 eq. and by addition to **L**-M(II) (1:1 molar ratio) solutions 0.5 eq. at a time of AE (Mg(II), Ca(II), Sr(II), Ba(II)) and RE (Eu(III), Dy(III), Gd(III)) ions up to 2.0 eq. In the case of **L1**-Cu(II), titrations with RE ions by adding 0.1 eq. at a time up to 2 eq. were performed.

^1^H and ^13^C-NMR spectra were acquired on a Bruker AVANCE 400 spectrometer (Billerica, MA, USA), operating at 400.13 and 100.61 MHz for ^1^H and ^13^C, respectively. For the spectra recorded in D_2_O, the peak positions are reported with respect to HOD (water-*d1*, δ = 4.75 ppm) for ^1^H–NMR spectra, while dioxane was used as reference standard in ^13^C NMR spectra (δ = 67.4 ppm). When necessary, pH was calculated as pH = pD − 0.40 [49]. ^1^H-^1^H and ^1^H-^13^C correlation experiments were performed to assign the signals. Chemical shifts (δ scale) are reported in parts per million (ppm values) relative to the characteristic peak of the solvent; coupling constants (*J* values) are given in hertz (Hz).

^1^H–NMR measurements to evaluate the stability of both **L1** and **L2** were performed at pH 4, 7.4 and 10. The analysis at pH 7.4 was carried out in H_2_PO_4_^−^/ HPO_4_^2−^ buffer.

### 3.2. EMF Measurements

Equilibrium constants for protonation and complexation reactions of the two ligands were determined by pH-metric measurements in degassed 0.15 mol dm^-3^ NaCl (for **L1**) or NMe_4_Cl (for **L2**) at 298 ± 0.1 K, using the fully automatic equipment that has already been described for **L1** [50,51,52,53] and **L2** [54], respectively.

EMF data were acquired with the PASAT computer program. The combined glass electrode was calibrated as a hydrogen concentration probe by titrating known amounts of HCl with CO_2_-free NaOH (for **L1**) or NMe_4_OH (for **L2**) solutions and determining the equivalent point by Gran’s method [55], which gives the standard potential E and the ionic product of water (pK_w_ = 13.73 ± 0.01 at 298.1 K in 0.1 mol dm^−3^ NaCl, for **L1**; p*K*_w_ = 13.83(1) at 298.1 K in 0.15 mol dm^−3^ NMe_4_Cl, for **L2**. *K*_w_ = [H^+^][OH^−^]).

At least three measurements (consisting of 100 data points for each) were performed for each system in the pH range 2–12, and all titrations were treated either as single sets or as separate entities, for each system; no significant variations were found in the values of the determined constants. In all experiments, ligand concentration ([**L**]) was 1 × 10^−3^ mol dm^−3^, while in complexation experiments a metal ion concentration of 0.8[**L**] was employed. The HYPERQUAD computer program [56] was used to process the potentiometric data. Distribution diagrams were obtained by using the Hyss program [57].

### 3.3. Synthesis

Compounds **L1** and **L2** were obtained following synthetic procedures analogous to those already described for malten and maltonis [39], and they are reported in Scheme 1. All chemicals were purchased in the highest quality commercially available; solvents were RP grade.

*N,N*′*-bis((3-hydroxy-4-pyron-2-yl)methyl)-1,4-piperazine Dihydroperchlorate (**L1**·2HClO_4_)*: α,α′-Azoisobutyronitrile (AIBN, 0.33 g, 0.002 mol) was added under an inert atmosphere to a refluxing and stirred solution of **2** [39] (2.3 g, 0.01 mol) and *N*-bromosuccinimide (NBS, 2.0 g, 0.011 mol) in CCl_4_ (50 mL). The resulting mixture was stirred and refluxed for 1 h (*R*_f_ = 0.85, hexane/ethyl acetate 1:1); the mixture was subsequently cooled at room temperature, and then filtered. The resulting yellow solution containing compound **3** was used without further purification, and added dropwise, in an inert atmosphere, to a stirred mixture of 1,4-piperazine (**4**, 0.43 g, 0.005 mol) and triethylamine (TEA, 1.41 mL, 0.011 mol) in THF (70 mL) at 0 °C. The reaction mixture was stirred at 0 °C for 48 h, and then filtered, resulting in a red-orange solution which was co-evaporated under vacuum several times with ethanol (3 × 100 mL). The resulting red oil residue was dissolved in ethanol (50 mL), and a 10% perchloric acid ethanolic solution was added dropwise under stirring until complete precipitation was achieved. The yellowish solid was filtered and recrystallized from a saturated sodium perchlorate aqueous solution to obtain **L1**·2HClO_4_ as a white solid (1.03 g, 36%): ^1^H–NMR (400 MHz, D_2_O pH = 12, 25 °C): δ = 7.80 (*H1*, d, *J* = 5.2 Hz, 2H), 6.30 (*H2*, d, *J* = 5.2 Hz, 2H), 3.57 (*H3*, s, 4H), 2.55 (*H4*, bs, 8H); ^13^C NMR (100 MHz, D_2_O pH = 12, 25 °C): δ = 181.5, 153.7, 153.4, 149.8, 112.4, 53.4, 51.5. MS (*m/z*) 335.3 (M + H). Elemental analysis calcd (%) for C_16_H_24_Cl_2_N_2_O_16_: C, 33.64; H, 4.23; N, 4.90; found: C, 34.6; H, 5.5; N, 4.9.

*N,N*′*,N*′*-tris((3-hydroxy-4-pyron-2-yl)methyl)-N-methylethylendiamine Hydroperchlorate (**L2**·HClO_4_):* α,α’-Azoisobutyronitrile (AIBN, 0.33 g, 0.002 mol) was added under an inert atmosphere to a refluxing and stirred solution of **2** [39] (4.5 g, 0.019 mol) and *N*-bromosuccinimide (NBS, 3.85 g, 0.021 mol) in CCl_4_ (90 mL). The resulting mixture was stirred and refluxed for 1 h (*R*_f_ = 0.85, hexane/ethyl acetate 1:1); the mixture was subsequently cooled at room temperature, and then filtered. The resulting yellow solution containing compound **3** was used without further purification, and added dropwise, in an inert atmosphere, to a stirred mixture of *N*-methylethylendiamine (**5**, 0.34 g, 0.0046 mol) and triethylamine (TEA, 2.7 mL, 0.02 mol) in THF (90 mL) at 0 °C. The reaction mixture was stirred at 0 °C for 48 h, and then filtered, resulting in a red-orange solution which was coevaporated under vacuum several times with ethanol (3 × 100 mL). The resulting red oil residue was dissolved in ethanol (50 mL), and a 10% perchloric acid ethanolic solution was added dropwise under stirring until complete precipitation was achieved. The yellowish solid was filtered and washed with warm CH_3_CN to obtain **L2**·HClO_4_ as a white solid (1.18 g, 45%): ^1^H–NMR (400 MHz, D_2_O pH = 12, 25 °C): δ = 7.72 (*H9*, d, *J* = 5.2 Hz, 2H), 7.69 (*H1*, d, *J* = 5.2 Hz, 1H), 6.22 (*H8*, d, *J* = 5.2 Hz, 2H), 6.24 (*H2*, d, *J* = 5.2 Hz, 1H), 3.68 (*H7*, s, 4H), 3.47 (*H3*, s, 2H), 2.60 (*H6*, m, 2H), 2.37 (*H5*, m, 2H), 2.06 (*H4*, s, 3H); ^13^C NMR (100 MHz, D_2_O pH = 12, 25 °C): δ = 181.5, 181.4, 153.6, 153.5, 153.3, 153.1, 150.8, 150.5, 112.6, 112.5, 54.1, 53.0, 51.5, 51.2, 41.7. MS (*m/z*) 447.4 (M + H). Elemental analysis calcd (%) for C_21_H_25_ClN_2_O_14_: C, 44.65; H, 4.46; N, 4.96; found: C, 44.4; H, 4.4; N, 5.2.

### 3.4. Biological Studies

The immortalized promonocytic leukemia (U937) cell line was obtained from American Type Culture Collection (ATCC). Cells were grown in RPMI 1640 (Lonza) supplemented with 10% fetal bovine serum (FBS), 1% penicillin-streptomycin and 1% glutamine in a humidified atmosphere at 37 °C, as previously described [35]. **L1** and **L2** were dissolved at 10 mM in distilled water as stock solutions, stored at −80 °C and subsequently diluted 1:10 as working solutions immediately before use. Treatments were carried out at the concentrations reported in the figures and cellular viability was evaluated after 48 h of treatment, by Trypan blue dye exclusion assay (using TC10 automatic cell counter—Bio-Rad) as previously described [58]. The data are reported as means (±SD) resulting from three independent experiments. The cytotoxic effect, on the other hand, was assessed considering the Trypan blue positive cells.

The DNA electrophoretic mobility assay was conducted incubating 1 μg of circular pLL3.7 plasmid DNA with the tested compound (at the final concentration of 4 mM each) in 20 μL of 10 mM Tris-HCl (pH 7.4) buffer. After incubation at the reported timing, DNA was separated by 0.8% AGE and then stained by GelRed^®^ (Biotium, Fremont, CA, USA) following the manufacturing instructions.

## 4. Conclusions

Two new maltol-based ligands, namely, *N*,*N*′-bis((3-hydroxy-4-pyron-2-yl)methyl)-1,4-piperazine (**L1**) and *N*,*N*′,*N*′-tris((3-hydroxy-4-pyron-2-yl)methyl)-*N*-methylethylendiamine (**L2**), were synthesized and characterized. Subsequent to the study of a similar compound (malten) showing intriguing biological activity and coordinating properties, ligands **L1** and **L2** were strategically designed to explore the effects of specific structural modifications on both biological and binding features.

The acid-base properties of both ligands have been studied; at physiological pH 7.4 two species are present in solution for both ligands, the zwitterionic **L** and the anionic H_-1_**L^-^** forms. At this pH value, **L1** shows higher stability to degradation (50% degradation time = 48 h) than **L2** (50% degradation time = 10 h).

Both ligands form stable complexes with first row transition metal ions M(II) (M(II) = Cu(II), Zn(II), Co(II)). While **L1** forms only stable mononuclear **L1**-M(II) species, among which the neutral [M(H_−2_**L1**)] is the prevalent one in a wide range of pH, including the neutral one, **L2** is also able to form metal complexes with more complex stoichiometries; namely, the binuclear and 3:2 (ligand to metal) species.

Analogously to malten, a N_2_O_2_ coordination environment around each M(II) ion can be suggested for **L1**, with two nitrogen atoms from the ethylendiamine and two oxygen atoms from two different maltol functions. The stabilities of **L1** complexes are lower than those of malten; this can be attributed to the insertion of the 1,4-piperazine, which makes more unfavorable the involvement of all N_2_O_2_ donor atoms in the stabilization of the M(II) ion with respect to the 1,2-dimethylendiamine of malten.

In **L2** mononuclear species, in addition to the diamine, the involvement of all the three maltol functions can be suggested in the coordination of Co(II), whereas only two maltol groups seem to be involved in the case of Cu(II) and Zn(II).

In the light of the previous studies on malten, the ability of the mononuclear species to behave as metallo-receptors towards AE and RE cations was investigated. In general, **L1**-M(II) systems are more affected than **L2**-M(II) systems by hard cations, suggesting that the insertion of the additional maltol moiety hampers the binding ability of the ligand. Moreover, RE ions perturb the UV-VIS absorption of the metal-coordinated ligands much more than AE ions do, due to the greater covalent character of the interaction with RE than AE.

In particular, only **L**-Cu(II) systems are perturbed by all AE cations, but for Mg(II) the interaction is small. All **L**-M(II) systems are instead perturbed by RE ions, **L1**-Cu(II) being the most deeply affected system. These data confirm the behavior already shown by malten, suggesting that notwithstanding the structural modifications introduced, **L1** and **L2** systems substantially maintain the peculiar binding properties towards hard cations.

The biological data support the hypothesis that **L1** and **L2** execute their biological activity via at least two distinct mechanisms. **L1**, notwithstanding its more rigid conformation, acts by a similar mechanism to malten, inducing complex structural alterations of DNA, not being, however, as effective as malten (even at the biological level). **L2**, which possesses an additional maltol group compared to malten, shows different behavior. It is indeed able to induce mainly single strand breaks (nicks) of DNA, and at longer time of exposure and to a lesser extent, double strand breaks also. For both **L1** and **L2** it can be excluded, based on previous studies, the hypothesis of an activity against DNA attributable to portions of the molecules tested [38]. Therefore, it came to light that the structural modifications brought on malten alter the mechanism of action and negatively impact the biological activity.

## Figures and Tables

**Table 1:** Protonation constants (log *K*) of **L1** and **L2** potentiometrically determined in 0.15 mol dm^−3^ NaCl and NMe_4_Cl, respectively, aqueous solution at 298.1 K.

Reaction	log *K*	
	L = L1	L = L2
H_−3_**L**^3−^ + H^+^ = H_−2_**L**^2−^	-	9.46(1)^1^
H_−2_**L**^2−^ + H^+^ = H_−1_**L**^−^	8.67(9)	8.33(1)
H_−1_**L**^−^ + H^+^ = **L**	7.77(7)	7.37(1)
**L** + H^+^ = H**L**^+^	5.88(8)	5.85(1)
H**L**^+^ + H^+^ = H_2_**L**^2+^	2.28(8)	1.86(2)

^1^ Values in parentheses are the standard deviations to the last significant figure.

**Table 2:** Equilibrium constants (log *K*) for the metal complex equilibria of **L1** and malten potentiometrically determined respectively in 0.15 mol dm^−3^ NaCl and NMe_4_Cl, aqueous solution at 298.1 K.

Reaction	log *K*					
	Co(II)		Cu(II)		Zn(II)	
	L = L1	L = Malten	L = L1	L = Malten [41]	L = L1	L = Malten
M^2+^ + H_−2_**L**^2−^ = [M(H_−2_**L**)]	7.46(9)^1^	10.35(1)	13.22(9)	17.53(1)	9.22(8)	10.29(1)
[M(H_−2_**L**)] + H^+^ = [M(H_−1_**L**)]^+^	-	5.95(2)	5.1(1)	3.84(1)	-	6.27(2)
[M(H_-1_**L**)]^+^ + H^+^ = [M**L**]^2+^	-	-	-	2.89(2)	-	-
[M(H_−2_**L**)] + OH^−^ = [M(H_−2_L)OH]^−^	-	2.92(4)	-	2.95(1)	-	2.95(4)

^1^Values in parentheses are the standard deviations to the last significant figure.

**Table 3:** Equilibrium constants (log *K*) for the metal complex equilibria of **L2** potentiometrically determined in 0.15 mol dm^−3^ NMe_4_Cl aqueous solution at 298.1 K.

Reaction	log *K*		
	Co(II)	Cu(II)	Zn(II)
M^2+^ + H_−3_**L**^3−^ = [M(H_−3_**L**)]^−^	12.88(1)^1^	18.25(2)	10.74(1)
[M(H_−3_**L**)]^−^ + H^+^ = [M(H_−2_**L**)]	5.71(1)	6.21(1)	8.58(2)
[M(H_−2_**L**)] + H^+^ = [M(H_−1_**L**)]^+^	5.57(2)	3.43(2)	5.57(2)
[M(H_−1_**L**)]^+^ + H^+^ = [M**L**]^2+^	-	3.41(2)	-
[M(H_−3_**L**)]^−^ + OH^−^ = [M(H_−3_L)OH]^2−^	-	3.79(3)	5.07(3)
[M(H_−3_**L**)OH]^2−^ + OH^−^ = [M(H_−3_**L**)(OH)_2_]^3−^	-	-	4.18(2)
M^2+^ + [M(H_−3_**L**)]^−^ = [M_2_(H_−3_**L**)]^+^	5.24(4)	7.63(3)	8.62(3)
[M_2_(H_−3_**L**)]^+^ + OH^−^ = [M_2_(H_3_**L**)OH]	3.57(3)	-	-
[M_2_(H_−3_**L**)OH] + OH^−^ = [M_2_(H_−3_**L**)(OH)_2_]^−^	2.47(4)	-	-
[M(H_−3_**L**)]^−^ + [M_2_(H_−3_**L**)]^+^ = [M_3_(H_−3_**L**)_2_]	-	5.02(5)	6.53(4)
[M_3_(H_−3_**L**)_2_] + OH^−^ = [M_3_(H_−3_**L**)_2_OH]^−^	-	4.59(6)	-
[M_3_(H_−3_**L**)_2_OH]^−^ + OH^−^ = [M_3_(H_−3_**L**)_2_(OH)_2_]^2−^	-	3.39(4)	-
[M_3_(H_−3_**L**)_2_(OH)_2_]^2−^ + OH^−^ = [M_3_(H_−3_**L**)_2_(OH)_3_]^3−^	-	3.22(5)	-

^1^ Values in parentheses are the standard deviations to the last significant figure.

## Supplementary Materials

The following are available online at https://www.mdpi.com/1420-3049/25/4/943/s1. Figure S1: ^1^H–NMR chemical shift of **L2** in aqueous solution as a function of pH, Figure S2: Trend of absorbance percentage variations of (a) **L1** and (b) **L2** at 275 nm at pH 4 (■) and 7.4 (●) and at 321 nm at pH 10 (▲) as a function of time. Figure S3: ^1^H–NMR spectra of **L1** recorded at different time intervals in buffered aqueous solution at pH 7.4. Figure S4: ^1^H–NMR spectra of **L2** recorded at different time intervals in buffered aqueous solution at pH 7.4, Figure S5: Distribution diagram of the species for the **L**-M(II) (**L** = malten, M(II) = (**a**) Zn(II), (**b**) Co(II)) system as a function of pH in aqueous solution; I = 0.15 mol dm^−3^ NMe_4_Cl, T = 298.1 K. [**L**] = [M(II)] = 1 × 10^−3^ mol dm^−3^. Figure S6: Distribution diagram of the species for the **L**-Cu(II) (**L** = **L2**) system as a function of pH in aqueous solution (I = 0.15 mol dm^−3^ NMe_4_Cl, T = 298.1 K. (a) [**L**] = 1 x 10^−3^ mol dm^−3^, [Cu^2+^] = 2 × 10^−3^ mol dm^−3^; (b) [**L**] = 1 × 10^−3^ mol dm^−3^, [Cu^2+^] = 1.5 × 10^−3^ mol dm^−3^. Figure S7: Distribution diagram of the species for the **L**-Zn(II) (**L** = **L2**) system as a function of pH in aqueous solution (I = 0.15 mol dm^−3^ NMe_4_Cl, T = 298.1 K. (a) [**L**] = 1 x 10^−3^ mol dm^−3^, [Zn^2+^] = 2 × 10^−3^ mol dm^−3^; (b) [**L**] = 1 × 10^−3^ mol dm^−3^, [Zn^2+^] = 1.5 × 10^−3^ mol dm^−3^. Figure S8: Distribution diagram of the species for the **L**-Co(II) (**L** = **L2**) system as a function of pH in aqueous solution (I = 0.15 mol dm^−3^ NMe_4_Cl, T = 298.1 K. [**L**] = 1 × 10^−3^ mol dm^−3^, [Co^2+^] = 2 × 10^−3^ mol dm^−3^. Figure S9: Absorption spectra in the UV range of the (a) **L1**-Cu(II) and (b) **L2**-Cu(II) system in aqueous solution at pH 7.4 obtained by adding amounts of Ca(II) up to 2 eq. with respect to **L**-Cu(II) system. [**L**] = [Cu^2+^] = 1.2 x 10^−5^ mol dm^−3^. Figure S10: Absorption spectra in the UV range of the **L2**-Cu(II) system in aqueous solution at pH 7.4 obtained by adding amounts of Dy(III) up to 2 eq. with respect to **L2**-Cu(II) system. [**L2**] = [Cu^2+^] = 1.2 × 10^−5^ mol dm^−3^.
