# Supplementary material for: Playing with Structural Parameters: Synthesis and Characterization of Two New Maltol-Based Ligands with Binding and Antineoplastic Properties
Source: Molecules. 2020 Feb 20;25(4):943. doi: 10.3390/molecules25040943 (PMC7070877; doi:10.3390/molecules25040943)
Supplement: Supplementary file 1 [file molecules-25-00943-s001.pdf]

Supporting Information

Playing with Structural Parameters: Synthesis and Characterization of Two New Maltol-Based Ligands with Binding and Antineoplastic Properties

Eleonora Macedi,* Daniele Paderni, Mauro Formica, Luca Conti, Mirco Fanelli, Luca Giorgi, Stefano Amatori, Gianluca Ambrosi, Barbara Valtancoli and Vieri Fusi*

Table of Contents

1. ¹H NMR studies on L2 1

2. Stability 2

3. Coordination of metal ions 5

1. ^{1}H NMR studies on L2

In Figure S1 the trend for the chemical shifts of the ^{1}H NMR resonances of **L2** as a function of pH is reported, while that for **L1** is reported in the main text, Figure 3 (see Figure 1 for atom labelling used in NMR assignments).

Analyzing **L2** (Figure S1), starting from alkaline pH and considering the pH range 12-8.5, where the H_{-3}**L2**$^{3-}$ and H_{-2}**L2**$^{2-}$ species are present in solution, it shows a marked downfield shift of all the aliphatic signals (H3, H4, H5 and H6) except for H7, meaning that the first protonation step involves the amine function bearing only one maltol moiety. The little downfield shift shown by H1 and H2 signals suggests the formation of a H-bond involving the same maltolate unit.

In the pH range 8.5-7.5, where the H_{-1}**L2**$^{-}$ species forms, no significant shifts of the aliphatic proton signals were observed, thus it can be deduced, in agreement with UV-VIS data, that a maltolate function is involved. The small shifts of H8 and H9 signals seem to suggest the engagement of a "twin" maltolate moiety.

In the pH range 7.5-6.5, where the **L2** species is prevalent in solution, the aromatic protons of the maltolate functions (H1, H2, H8, H9) downfield shift along with the resonances of the protons close to both amine functions. Considering also the UV-VIS data, the protonation of the remaining "twin" maltolate fragment can be suggested, together with the formation of H-bonds involving the diamine scaffold.

In the pH range 6.5-4, where the H**L2**$^{+}$ species is prevalent in solution, the aromatic H1 and H2 shift downfield, along with aliphatic H3, H4, H5 and H6 signals suggesting that this protonation step occurs on the maltolate function linked to the methylated amine function with consequent disruption of H-bond involving this fragment.

Finally, at pH < 4, where the fully protonated H_2**L2**$^{2+}$ species is present in solution, the H7 resonance exhibits the main shift supporting that the last protonation step mainly involves the amine moiety.

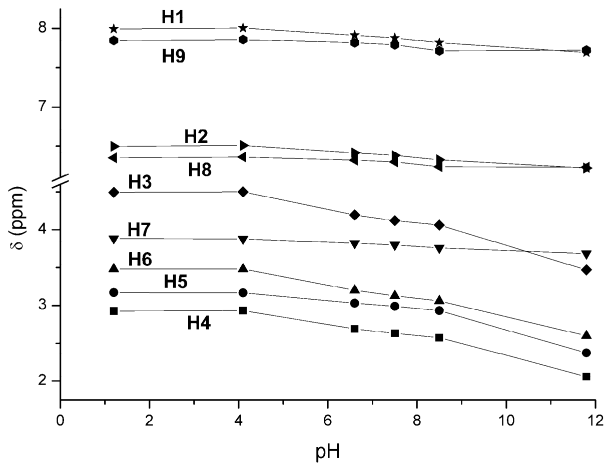

Figure S1. ^{1}H NMR chemical shift of **L2** in aqueous solution as a function of pH.

2. Stability

The trend of the absorbances at 275 and 321 nm of **L1** and **L2** at the tested pH values (Fig. S1) highlights that over five days an amount of sample comprised between 15 and 19% of **L1** is lost at all pH values: overall, the ligand is slightly more stable at pH 7.4 than at acid and basic pH values.

In the case of **L2**, the decomposition process is higher at pH ≥ 7, where an amount of sample ranging from 36 to 40% is lost in five days. At pH 4, where the HL^+ species is present in solution, only 2.4% of **L2** is lost over the total period: as a consequence, it can be said that **L2** is stable at acidic pH for at least five days.

Overall, **L2** decomposes far more than **L1** at pH values higher than 7 while much less at pH 4.

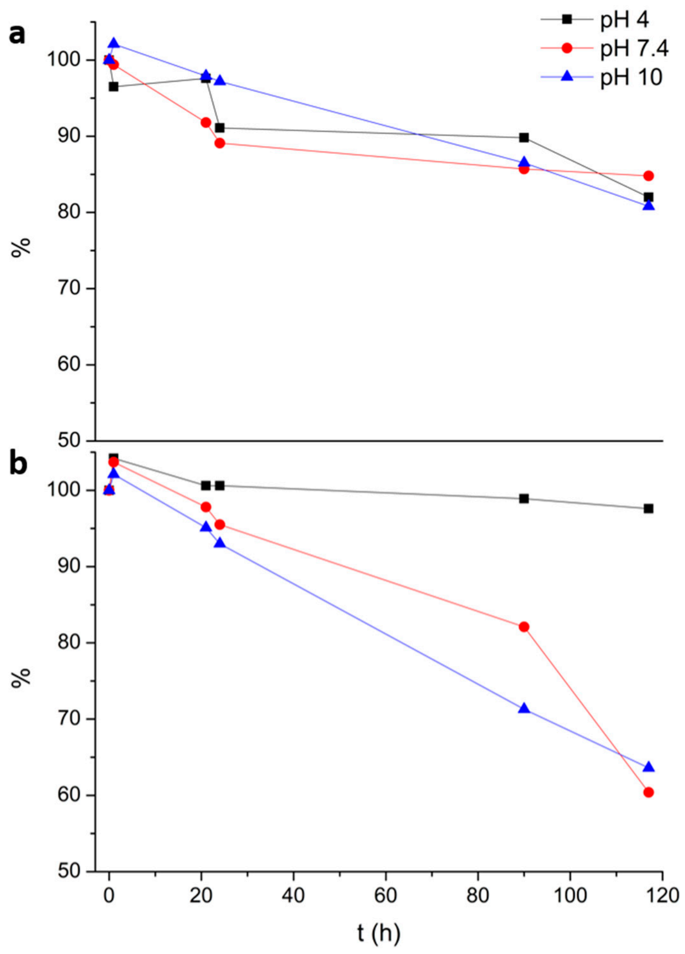

Figure S2. Trend of absorbance percentage variations of **a**) **L1** and **b**) **L2** at 275 nm at pH 4 (■) and 7.4 (●) and at 321 nm at pH 10 (▲) as a function of time.

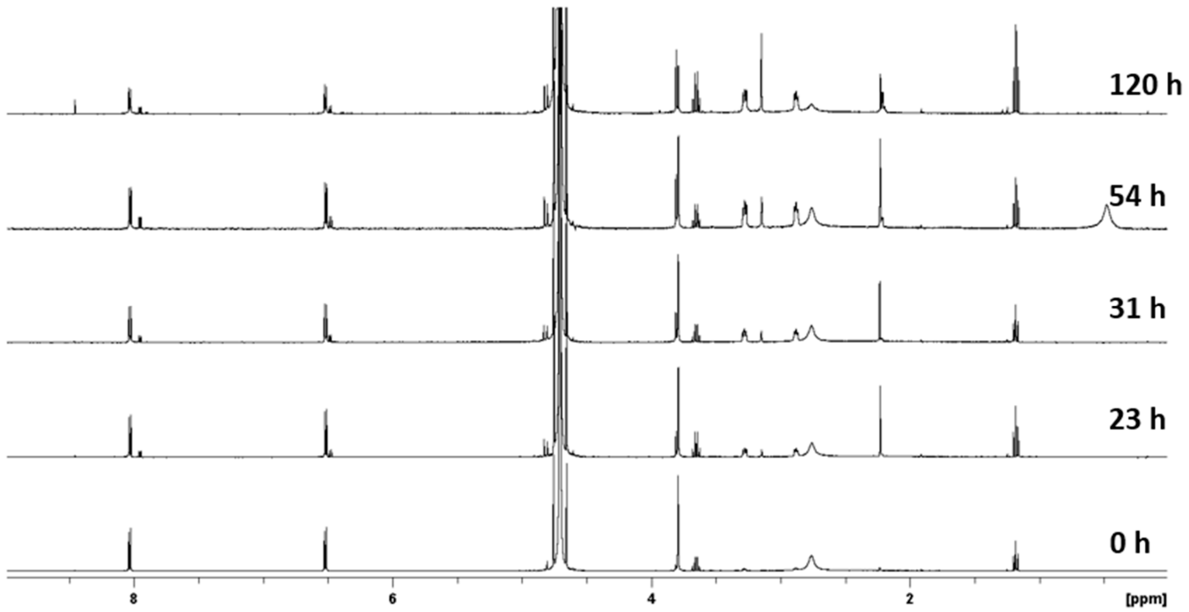

Figure S3. ^{1}H NMR spectra of **L1** recorded at different time intervals in buffered aqueous solution at pH 7.4.

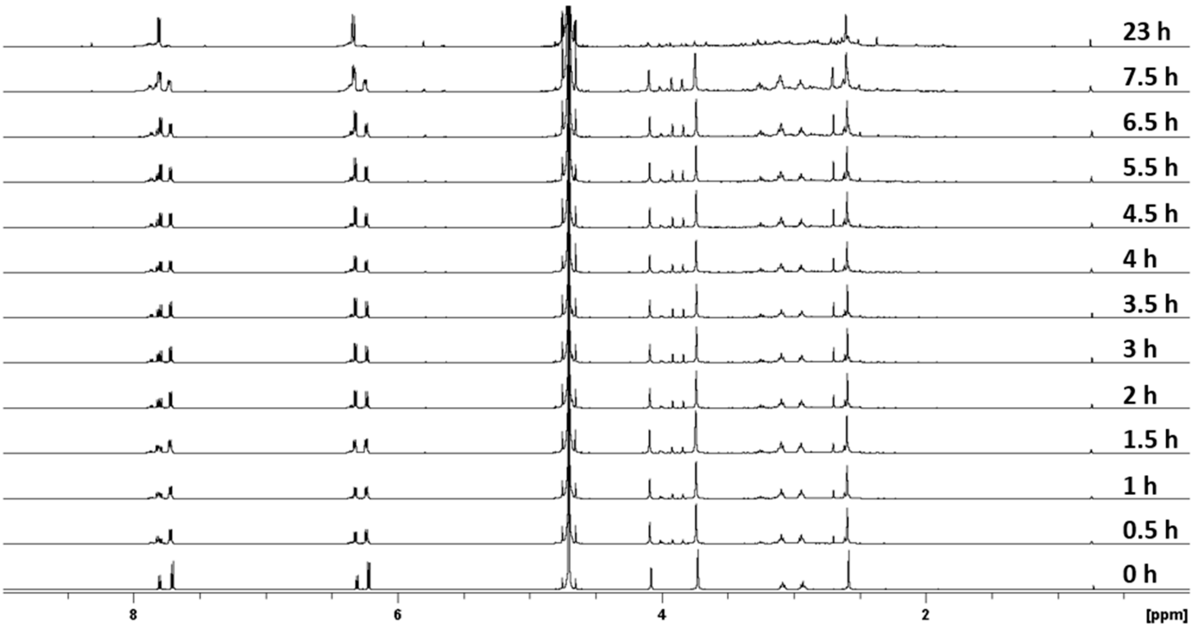

Figure S4. ^{1}H NMR spectra of **L2** recorded at different time intervals in buffered aqueous solution at pH 7.4.

3. Coordination of metal ions

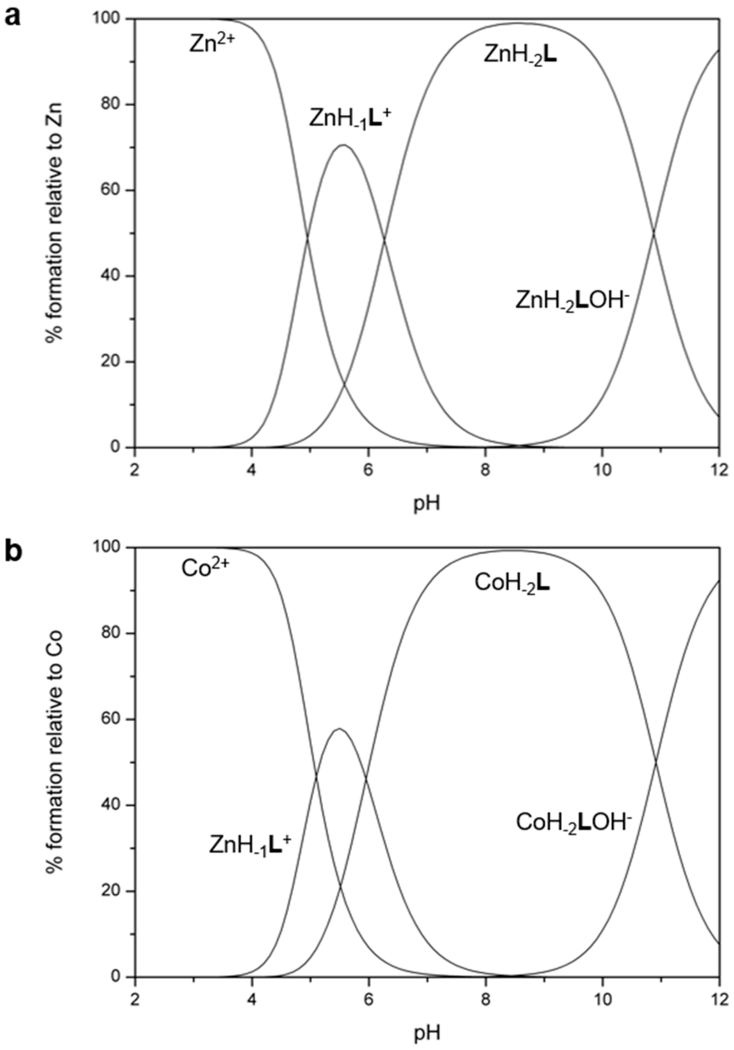

Figure S5. Distribution diagram of the species for the **L**-M(II) (**L** = Malten, M(II) = (**a**) Zn(II), (**b**) Co(II)) system as a function of pH in aqueous solution; I = 0.15 mol dm^{-3} NMe_4Cl, T = 298.1 K. [**L**] = [M(II)] = 1 x 10^{-3} mol dm^{-3}.

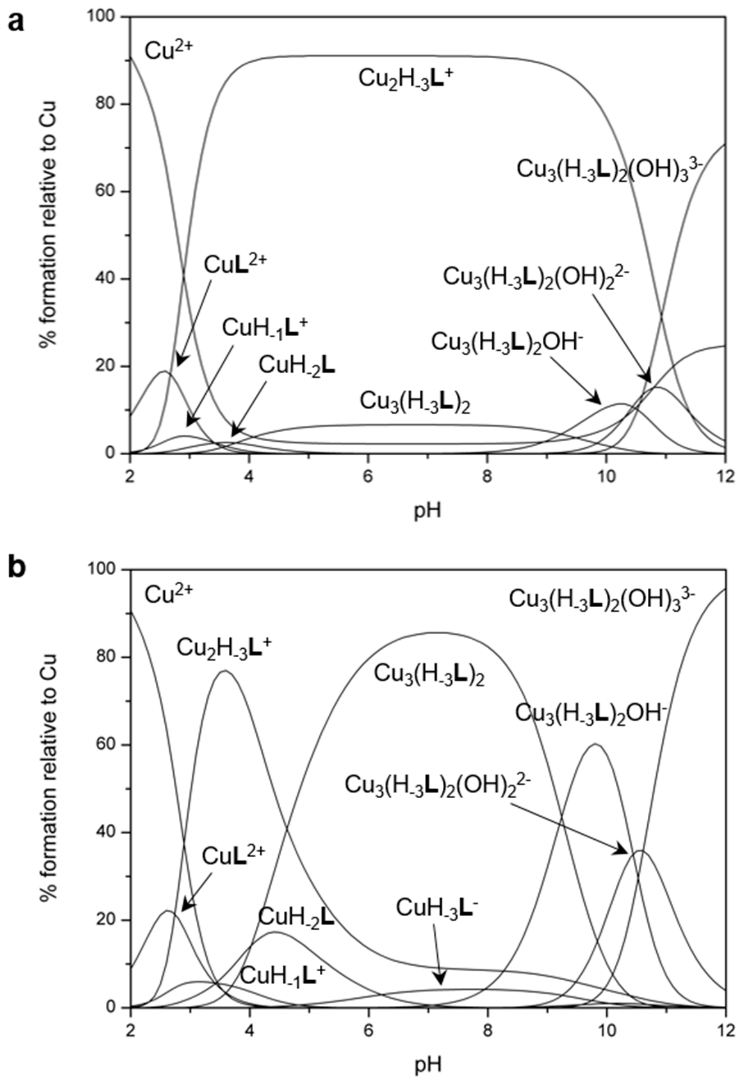

Figure S6. Distribution diagram of the species for the **L**-Cu(II) (**L** = **L2**) system as a function of pH in aqueous solution (I = 0.15 mol dm^{-3} NMe_4Cl, T = 298.1 K. **a)** [**L**] = 1 x 10^{-3} mol dm^{-3}, [Cu^{2+}] = 2 x 10^{-3} mol dm^{-3}; **b)** [**L**] = 1 x 10^{-3} mol dm^{-3}, [Cu^{2+}] = 1.5 x 10^{-3} mol dm^{-3}.

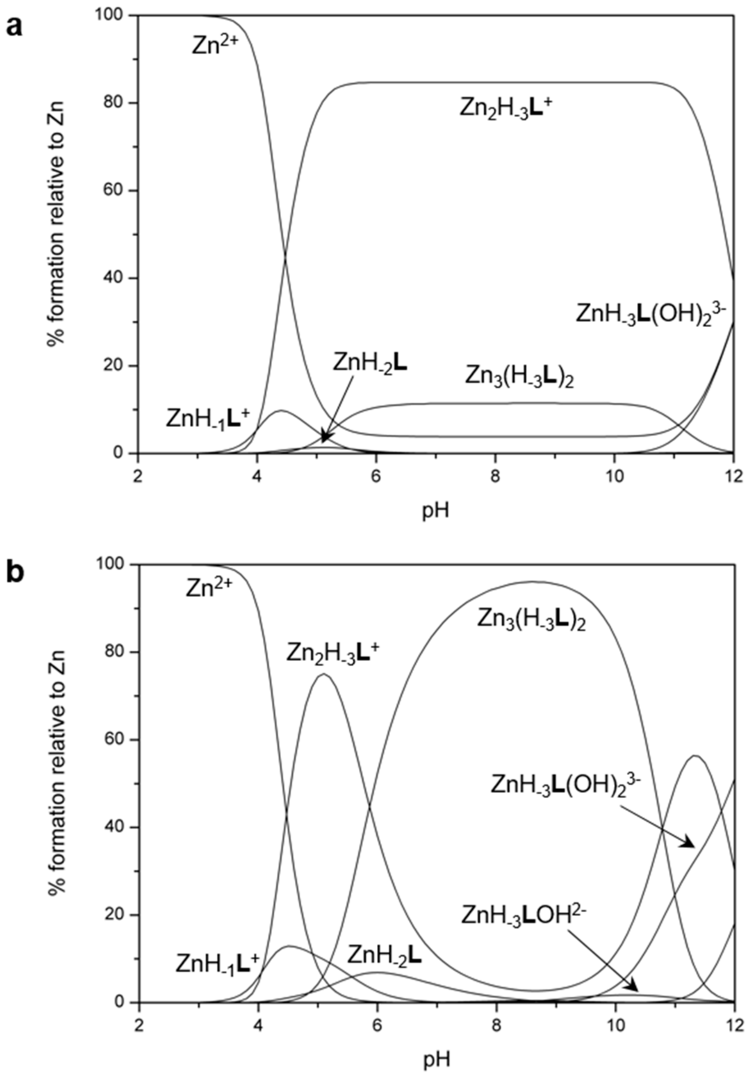

Figure S7. Distribution diagram of the species for the **L**-Zn(II) (**L** = **L2**) system as a function of pH in aqueous solution (I = 0.15 mol dm^{-3} NMe_4Cl, T = 298.1 K. **a)** [**L**] = 1 x 10^{-3} mol dm^{-3}, [Zn^{2+}] = 2 x 10^{-3} mol dm^{-3}; **b)** [**L**] = 1 x 10^{-3} mol dm^{-3}, [Zn^{2+}] = 1.5 x 10^{-3} mol dm^{-3}.

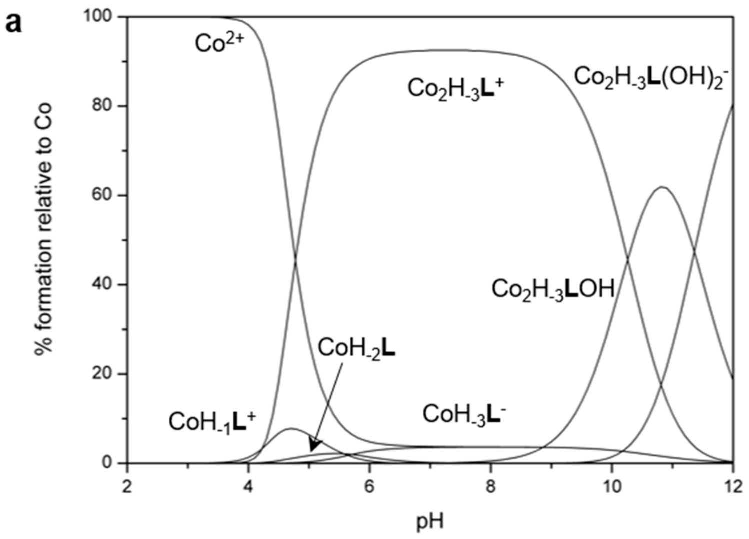

Figure S8. Distribution diagram of the species for the **L**-Co(II) (**L** = **L2**) system as a function of pH in aqueous solution (I = 0.15 mol dm^{-3} NMe_4Cl, T = 298.1 K. [**L**] = 1 x 10^{-3} mol dm^{-3}, [Co^{2+}] = 2 x 10^{-3} mol dm^{-3}.

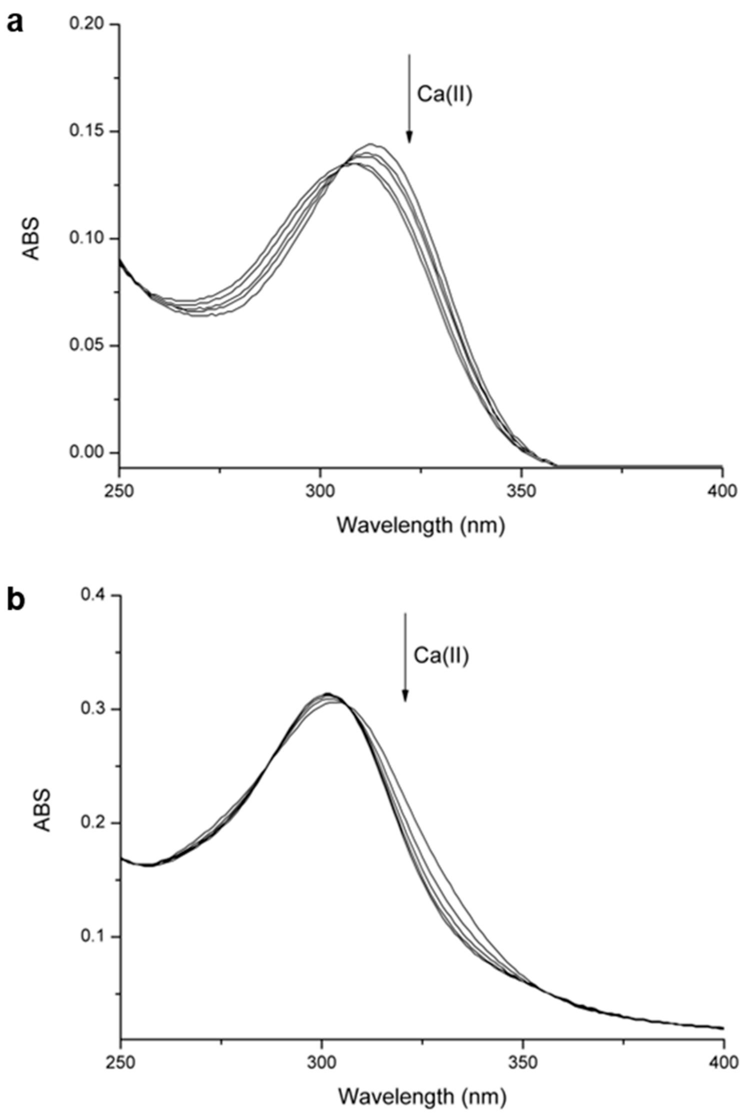

Figure S9. Absorption spectra in the UV range of the a) **L1**-Cu(II) and b) **L2**-Cu(II) system in aqueous solution at pH 7.4 obtained by adding amounts of Ca(II) up to 2 eq. with respect to **L**-Cu(II) system. [**L**] = [Cu^{2+}] = 1.2 x 10^{-5} mol dm^{-3}.

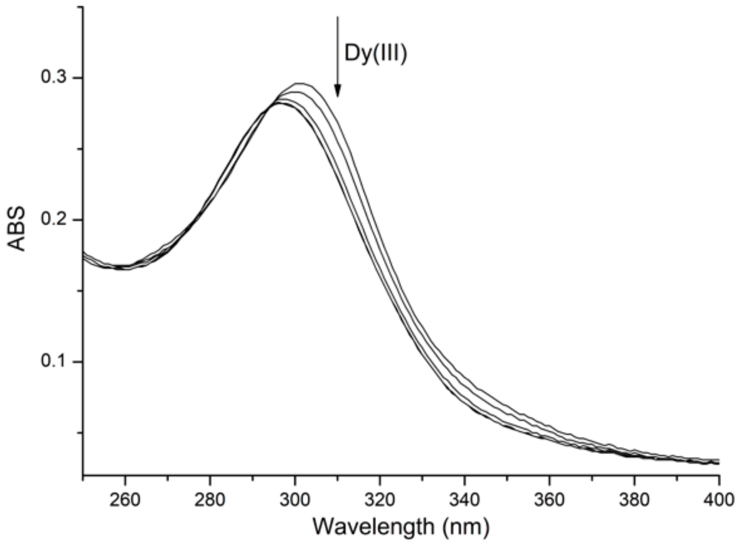

Figure S10. Absorption spectra in the UV range of the **L2**-Cu(II) system in aqueous solution at pH 7.4 obtained by adding amounts of Dy(III) up to 2 eq. with respect to **L2**-Cu(II) system. [**L2**] = [Cu^{2+}] = 1.2 x 10^{-5} mol dm^{-3}.
